# Uncovering transcriptional reprogramming during callus development in soybean: insights and implications

**DOI:** 10.3389/fpls.2023.1239917

**Published:** 2023-08-04

**Authors:** Joo-Seok Park, Yoram Choi, Min-Gyun Jeong, Yeong-Il Jeong, Ji-Hyun Han, Hong-Kyu Choi

**Affiliations:** ^1^ Department of Applied Bioscience, Dong-A University, Busan, Republic of Korea; ^2^ Department of Molecular Genetics, Dong-A University, Busan, Republic of Korea

**Keywords:** transcriptome, soybean callus, wounding, phytohormones, transcription factors, RNA-sequencing

## Abstract

Callus, a valuable tool in plant genetic engineering, originates from dedifferentiated cells. While transcriptional reprogramming during callus formation has been extensively studied in *Arabidopsis thaliana*, our knowledge of this process in other species, such as *Glycine max*, remains limited. To bridge this gap, our study focused on conducting a time-series transcriptome analysis of soybean callus cultured for various durations (0, 1, 7, 14, 28, and 42 days) on a callus induction medium following wounding with the attempt of identifying genes that play key roles during callus formation. As the result, we detected a total of 27,639 alterations in gene expression during callus formation, which could be categorized into eight distinct clusters. Gene ontology analysis revealed that genes associated with hormones, cell wall modification, and cell cycle underwent transcriptional reprogramming throughout callus formation. Furthermore, by scrutinizing the expression patterns of genes related to hormones, cell cycle, cell wall, and transcription factors, we discovered that auxin, cytokinin, and brassinosteroid signaling pathways activate genes involved in both root and shoot meristem development during callus formation. In summary, our transcriptome analysis provides significant insights into the molecular mechanisms governing callus formation in soybean. The information obtained from this study contributes to a deeper understanding of this intricate process and paves the way for further investigation in the field.

## 1 Introduction

Plant cells exhibit a notably superior capacity for regeneration when compared to animal cells. This remarkable regenerative potential arises from their developmental plasticity, which is evident through the process of dedifferentiation and transdifferentiation. These abilities are prominently shown in phenomena like callus formation and grafting. Particularly, when plants are exposed to stresses such as wounding or pathogen infection, they can generate unorganized masses of cells known as callus (Ikeuchi et al., 2013). Initially, the term “callus” described a substantial accumulation of cells producing callose at a wound site. However, its current definition encompasses disorganized clusters of cells that arise from alterations in cellular state, such as excessive proliferation and the transdifferentiation of specialized cells (Fehér, 2019). Callus can be developed from a single somatic cell that has already differentiated and may possess totipotency, which means that each callus cell has the potential capability of regenerating an entire plant via somatic embryogenesis, a process similar to embryonic development in non-zygotic cells (Ikeuchi et al., 2019; Bidabadi and Jain, 2020). During this process, the fate of cells can be reprogrammed depending on the types of imposed stresses and the relative concentrations of phytohormones. The innate regenerative capability of plants has made it possible to introduce functionally useful genes into a callus, usually using *Agrobacterium*, and subsequently produce beneficial substances in plants, making it a valuable tool in the field of plant-derived biotechnologies (Belide et al., 2017; Hwang et al., 2019; Cordeiro et al., 2023).

Auxin and cytokinin are two phytohormones that play central roles in controlling callus development. The ratios of these hormones are believed to determine the direction of the cell’s developmental fate (Skoog and Miller, 1957). Generally, a higher concentration of auxin relative to cytokinin stimulates root formation, while a reversed ratio favors shoot regeneration. *In vitro*, callus induction is typically conducted on callus-inducing media (CIM) with a proper balance between auxin and cytokinin. The gene expression pattern of CIM-induced callus is known to resemble root meristem development (Sugimoto et al., 2010). Auxin promotes callus formation by activating genes such as lateral organ boundary domain 16 (LBD16), LBD17, and LBD18, which are mediated by auxin response factors (ARFs) like ARF7 and ARF19 (Okushima et al., 2007). LBDs, in turn, activate the expression of genes that promote cell proliferation and modify cell wall structures. Unlike the CIM-induced pathway, wounding can induce callus formation through a pathway that does not follow root initiation. Wounding activates the central regulator wound induced dedifferentiation 1 (WIND1) and its paralogous genes WIND2, WIND3, and WIND4 (Iwase et al., 2011). The wounding signal triggers cytokinin biosynthetic pathway genes such as isopentenyl transferase 3 (IPT3) and lonely guy (LOG1, LOG4, and LOG5) (Ikeuchi et al., 2017). This, in turn, activates type-B *Arabidopsis* response regulator 1 (ARR1) and ARR12 involved in cytokinin signaling, leading to cell cycle reentry by cyclin D3;1 (CYCD3;1).

Soybean [*Glycine max* (L.) Merr.], a legume crop, possesses a diploidized paleopolyploid genome that consists of 20 chromosome pairs that underwent the latest whole genome duplication (WGD) about 13 million years ago (MYA). It is widely recognized as a major source of dietary protein and oil for humans. Soybean, along with other legumes, is renowned for its ability to fix nitrogen through symbiosis with Rhizobium bacteria. The whole genome of soybean, estimated to be 1.115 gigabase (Gb) in size, has been fully sequenced, and a recent transcriptome analysis revealed 54,132 confidently expressed genes. The prevalence of paralogous genes and the existence of a substantial proportion of multi-gene families (~75%) in the soybean genome is largely attributed to its WGD nature (Schmutz et al., 2010; Wang et al., 2014).

This study aims to uncover the biological mechanism underlying soybean callus induction and developmental process. To achieve this goal, the transcriptome changes associated with the soybean callus formation process were comprehensively analyzed. This involves dividing the development of soybean callus into seven time points (0, 1, 4, 7, 14, 28, and 42 days) to observe how the transcriptome is affected by morphological changes over time. To confirm the physiological mechanisms related to soybean callus formation, we analyzed the transcriptomic dynamics using various methods such as differentially expressed genes (DEGs), c-mean clustering, and gene ontology (GO) enrichment for genes related to hormones, cell wall, cell cycle, and transcription factors.

## 2 Materials and methods

### 2.1 Plant materials and callus induction

Callus induction was performed using seeds of soybean (*G. max* L. Merr, cv. Kwang-an). The surface of seeds was sterilized using chlorine gas, which was generated by combining 5ml of 12N HCl and 45ml of 12% hypochlorite solution in a dessicator for a duration of 16 hours. After that, the seeds were subjected to incubation with 12ml of deionized water (DIW) per 150 seeds. After 24 hours of moist incubation, the seeds were washed with a 1% (v/v) mild bleach solution, followed by three additional washes with autoclaved DIW. The seeds were further incubated for one day in a 50 ml conical tube filled with water. After soaking, the seeds were halved using scalpel (#11 blade), and the embryonic axes were removed. To induce the callus formation, the divided seeds underwent surface scratching in multiple directions, with each seed being scratched 10 times. Additionally, the junction of the hypocotyl and cotyledon was scratched perpendicular to the cotyledon (Jiang et al., 2010). Following that, the divided seeds were implanted with CIM (Gamborg’s B5 medium) containing 3 mM MES, 3% (w/v) sucrose, 8 μg/ml of 2,4-D, 1 μg/ml of BA, and 250 μg/ml of cefotaxime. To activate callus induction, the plants were grown in darkness (24 hours dark) at 28 °C. To prevent nutrient depletion, the plants were sub-cultured every two weeks. Callus formation, consisting of meristem along with a small portion of cotyledon and hypocotyl, was observed at various time points after wounding; 1, 4, 7, 14, 28 and 42 days after wound (DAW). In contrast, a control sample at 0 DAW remained unwounded. All samples were collected at room temperature, rapidly frozen in liquid nitrogen, and stored at -80 °C until RNA extraction.

### 2.2 RNA extraction and sequencing

Total RNA was extracted from the explants at 0, 1, 4, 7, 14, 28, and 42 DAW. For RNA sequencing, the RNA samples were pooled from 10 explants at each time point and isolated using the RNeasy Plant Mini Kit (Qiagen, Hilden, Germany). The RNA integrity was evaluated through agarose gel electrophoresis, where the ratio of the large subunit and small subunit of rRNA was examined. The analysis revealed consistent intensity across samples, indicating the preservation of RNA integrity. Moreover, the purity of RNA samples was assessed by measuring the A260/A280 ratio using a spectrophotometer (Implen, Munchen, Germany). The obtained results confirmed that both the integrity and purity of the RNA were well-maintained, as the A260/A280 ratio showed minimal deviation from expected range of 1.8~2.0. Sequencing libraries were prepared for each sample using 2 μg of total RNA and the TrueSeq Stranded Total RNA LT Prep Kit (Plant) (Illumina, CA, USA) following the manufacturer’s instructions. Paired-end reads for the seven samples were generated using the Illumina NovaSeq 6000 platform (Macrogen, Seoul, ROK) with three biological replicates.

### 2.3 Transcriptome analysis

The sequencing reads generated from RNA sequencing were aligned to the soybean reference genome (Wm82.a2.v1) by utilizing the HISAT2 aligner^1^ (Kim et al., 2019) with the default settings. The raw read counts of each gene were determined using HTSeq^2^ (Anders et al., 2015) with the options “–mode union –stranded no –format bam”. The DESeq2 R package (Love et al., 2014) was used to calculate the normalized count and identify DEGs at each time point compared to day 0, using the criteria of |log2fold change| ≥ 2 and false discovery rate (FDR) adjusted p-value < 0.05. To analyze the relationships among samples, principal component analysis (PCA) was performed using the regularized logarithms (rlog) of read counts calculated by DESeq2. The Venn diagram and heatmap of DEGs at each time point were generated using InteractiVenn^3^ (Heberle et al., 2015) and the pHeatmap R package (Kolde, 2015), respectively. Mfuzz R package (Kumar and Futschik, 2007) was used to cluster the expression levels of all significantly changed DEGs, based on c-mean clustering. The optimal fuzzifier m value, which prevented clustering of random gene expression, was estimated as 1.53.

### 2.4 Gene ontology and KEGG enrichment analysis

The enrichment analysis of GO and KEGG pathways was performed using the clusterProfiler R package (Wu et al., 2021). The analysis was conducted with the criterion of p-value < 0.005 to identify the functional categories among the DEGs. The annotation information and GO data were obtained from Phytozome^4^ (Goodstein et al., 2012) and PlantRegMap^5^ (Tian et al., 2019), respectively. The organism database used in GO enrichment analysis was generated using the AnnotationForge R package. The enzyme information of the induced genes was obtained from the locus mapping table in TGIL platform, as the Phytozome and KEGG gene IDs did not match for KEGG term enrichment. The visualization of enriched terms for each cluster was generated using the dot plot method in clusterProfiler.

### 2.5 Analysis of transcriptional regulation

We obtained information on all soybean transcription factors (TFs) and their interactions from PlantTFDB v4.0^6^ (Jin et al., 2017) and PlantRegMap, respectively. To identify significantly enriched TF families, we used the enrichment method in PlantRegMap, which revealed a total of 18 enriched families.

### 2.6 Real-time quantitative PCR analysis

To validate the expression patterns obtained from RNA sequencing, we selected nine genes known to be involved in callus generation in *A. thaliana* (Ikeuchi et al., 2017). Orthologous genes in soybean were identified using the TGIL platform^7^. One microgram of total RNA was reverse transcribed into cDNA using the SuPrimeScript RT Premix with oligo (dT) 2X kit (GeNet Bio, Korea). qRT-PCR was performed with the following steps: pre-denaturation at 95 °C for 30 seconds, denaturation at 95 °C for 5 seconds, and extension at 60 °C for 40 seconds. Denaturation and extension were repeated for 40 cycles, and the melt curve was obtained by increasing the temperature incrementally by 0.5 °C from 65 °C to 95 °C at the final step. The delta-delta Ct method (Gao et al., 2017) was used for the relative quantification of gene expression patterns, with tubulin beta 3 (TUBB3) selected as the control gene for normalization (Rao et al., 2013). All primers were designed automatically using an in-house Python program and are listed in **Table S1**. PCR amplifications were performed with three biological replicates and three technical replicates.

## 3 Result

### 3.1 Morphological alterations during the callus development

**Figure 1:**
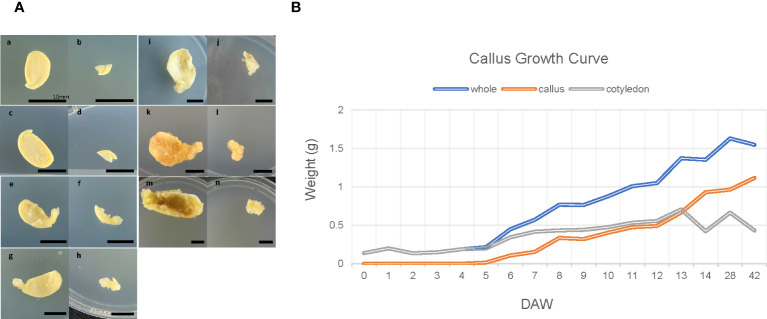
Morphological changes and growth curve during callus induction of soybean. **(A)** Morphological changes during callus formation. The left half of each image shows seed sections, with samples for RNA-seq (including meristem, part of hypocotyl, and cotyledon) on the right (samples labeled b, d, f, h, j, l, and n). Samples a and b were not wounded and served as control samples, while samples c-n were scratched using a surgical blade and incubated on CIM for varying durations. The time points captured in the images are as follows: 1DAW (days after wounding; c and d), 4DAW (e and f), 7DAW (g and h), 14DAW (i and j), 28DAW (k and l), 42DAW (m and n). Each bar in the images corresponds to 10mm. **(B)** Growth curve during callus development. The plot shows the callus growth curve, with ten explants pooled at each time point and the weight of each callus sample measured. The blue line represents actual samples used for RNA-sequencing (corresponding to samples b, d, f, h, j, l, and n in panel **A**), while the orange line represents only callus collected by raking up using a blade, and the gray represents a part of hypocotyl and cotyledon excluding callus.

Significant morphological changes were observed during callus formation (**Figure 1**), which can be categorized into three stages. The first stage, which lasts from 0 DAW to 4 DAW samples (**Figure 1A**a–f), is known as the lag phase. During this phase, the callus is visible (**Figures 1A**e, f), and its mass can be measured for the first time (**Figure 1B**). The second stage, which begins on day 4, is the log phase. At this stage, the callus grows actively and rapidly, appearing translucent yellow, fragile, and watery. The callus continues to grow fresh until days 7-8 (**Figures 1A**g, h, B) as the callus cell divides actively and rapidly. The final stage is the stationary phase, which begins after days 13-14 (**Figures 1A**i–n). At this stage, half-seed still grows, but the texture of the callus changes completely. The surface becomes stiff, dark, and opaque (**Figures 1A**k–n). The callus appears to be dying, while the cotyledon grows quickly and becomes senescent.

### 3.2 Genome-wide gene expression patterns during callus formation

**Figure 2:**
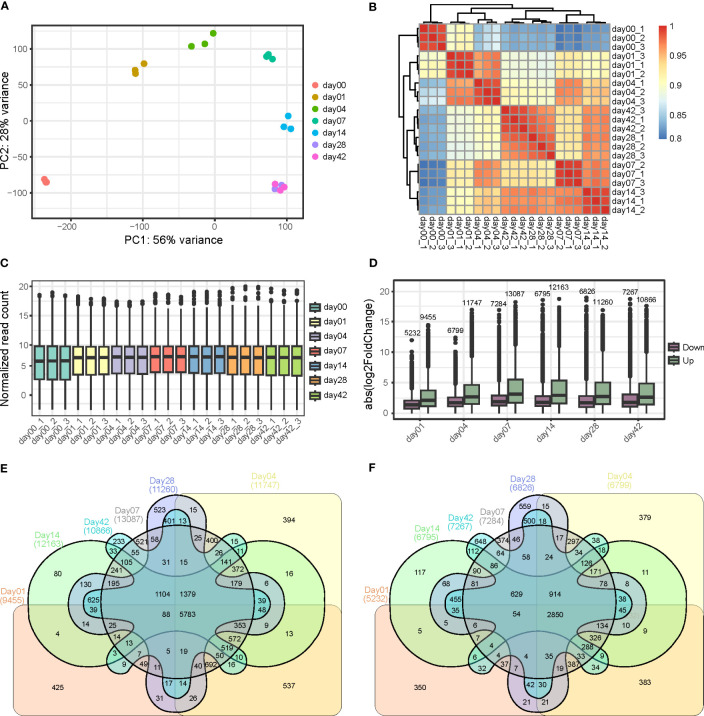
Analysis of sample similarity and differentially expressed genes during callus formation. **(A)** Principal component analysis (PCA) of RNA-seq from each day during callus formation. **(B)** Heatmap displaying the correlation between samples. **(C)** Distribution of expressed genes in different samples with replicates. **(D)** Distribution of differentially expressed genes in the various samples compared with day 0. **(E)** Comparison of up-regulated genes at different time points. **(F)** Comparison of downregulated genes at different stages.

In order to perform a comprehensive transcriptome analysis of soybean callus formation, we collected a total of 21 samples at seven distinct time points: 0, 1, 4, 7, 14, 28, and 42 days. Each time point consisted of three biological replicate to ensure a robust statistical analysis. This design allowed us to capture the global transcriptome changes occurring during the process of soybean callus formation. The RNA sequencing was conducted using the Illumina NovaSeq 6000 platform, which generated a total of 661 Gb obtained from paired-end reads. After filtering low-quality and adaptor-polluted reads, 84.76% of the clean reads with >Q30 Phred score were mapped to the soybean reference genome (Gmax.v.2.0). The average ratio of concordant pair alignment was 96.31% (ranging from 90.51% to 97.62%) (**Table S2**). Principal components analysis (PCA) was conducted to evaluate the similarities between individual samples. The results showed that samples from different time points were clearly separated along the first and second principal components, which explained 56% and 28% of the total variation, respectively (**Figure 2**). Moreover, the biological replicates at each time point were clustered together, indicating the high reproducibility of the experiment. Additionally, the dot plot revealed a significant difference in sample similarity between 0, 1, 4, 7, and 14 days based on PC1, and the PC2 value gradually increased until day 7 and then decreased. Thus, we hypothesized that genes related to callus formation, cell division, and response to hormone and wounding stress were strongly expressed up to 7 days, while genes involved in plant growth and senescence were activated thereafter. To validate this hypothesis, we conducted GO enrichment analysis using the top 1000 genes associated with PC1 and PC2, respectively. Our results showed that GO terms related to stress response and senescence were overrepresented in PC1, whereas GO terms involved in cell wall modification and response to auxin were highly enriched in PC2 (**Figure S1**). Based on these findings, we could conclude that genes associated with stress response were significantly activated by wounding stress on day 1, followed by genes related to callus formation up to day 7, and genes involved in growth and senescence thereafter.

DESeq2 was utilized to compute expression values for each sample. From the total of 42,466 genes that had normalized expression values greater than 3 in at least one sample, DEGs were identified by comparing each time point sample to day 0 (|log2fold change| >= 1 and FDR < 0.05) (**Table S3**). A total of 27,639 genes exhibited perturbations in their expression pattern during callus formation. Of these, 9,455, 11,747, 13,087, 12,163, 11,260, and 10,866 genes were up-regulated for 1, 4, 7, 14, 28, and 42 days, respectively, while 5,232, 6,799, 7,284, 6,795, 6,826, and 7,267 genes were down-regulated for the same respective time points (**Figure 2**). To validate the expression values obtained from RNA-seq, nine genes from the up-regulated genes were selected for qRT-PCR analysis, and the results were comparable to those obtained from RNA-Seq. This finding, presented in **Figure S2**, supports the robustness of the RNA-Seq experiment.

### 3.3 Clustering and GO enrichment analysis of callus development-related genes

**Figure 3:**
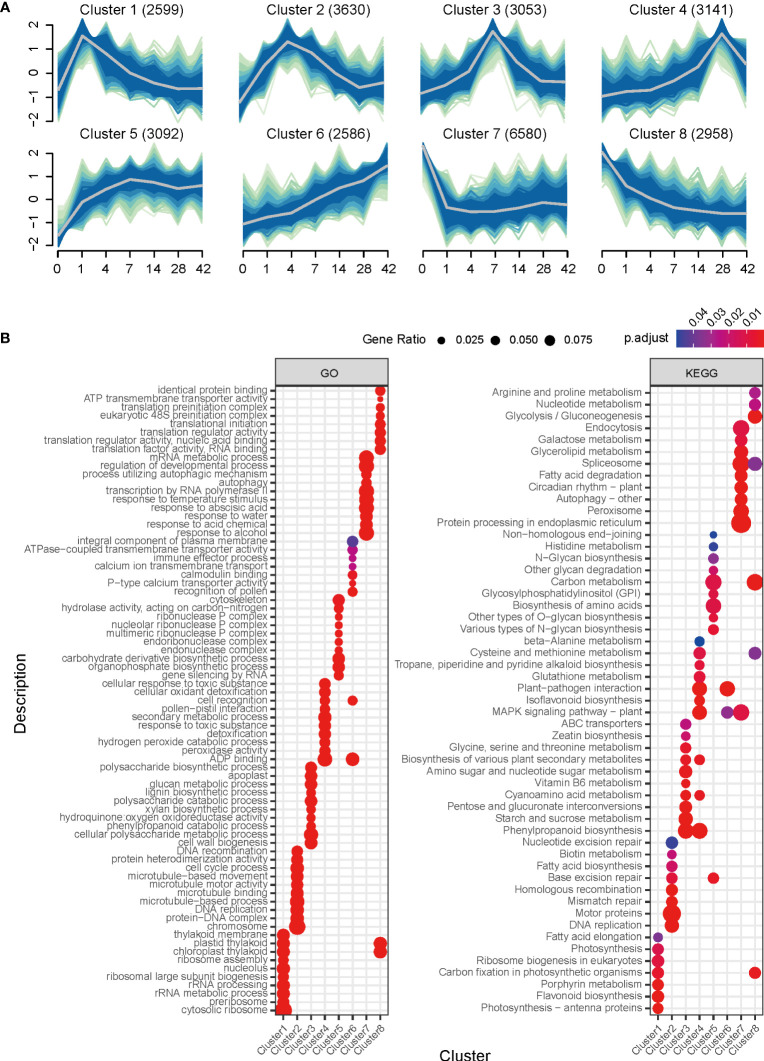
Clustering and GO enrichment analysis of RNA-seq data during callus formation. **(A)** Clustering of differentially expressed genes. The eight clusters, based on time-series expression patterns at Day 1, Day 4, Day 7, Day 14, Day 28, and Day 42 during callus formation, were grouped by induced genes. The fuzzy c-means (FCM) clustering algorithm, implemented in the Mfuzz R package, was used to cluster genes based on standardized expression change values. A membership value between 0 and 1 is assigned to each gene, and cluster cores are highlighted with gray line. **(B)** GO and KEGG Pathway enrichment analysis for clusters. The dot plot shows the top 15 overrepresented GO terms of biological process, cell component, and molecular function identified using clusterProfiler with differentially expressed genes for each cluster.

To better understand the functional meaning of transcriptomic changes during callus formation, the Mfuzz R program was used to group the 27,639 induced genes into eight clusters based on their time-series expression patterns (**Figure 3A**; **Table S4**). These clusters were then subjected to downstream analysis, such as GO enrichment, to assign functional criteria to each cluster (**Table S5**).

Cluster 1, which consisted of 2,599 genes, showed a rapid increase in expression at day 1 followed by a gradual decrease. This cluster mainly contained genes expressed in response to wounding and callus induction. The results of the GO enrichment analysis indicated the involvement of biological processes such as auxin-activated signaling pathway, regulation of hormone levels, and lateral root formation (**Figure 3**; **Table S5**), suggesting that callus initiation process responsive to wounding and auxin was turned on at day 1.

Cluster 2, consisting of 3,630 genes, exhibited strong expression on days 4 and 7, which gradually decreased over time. GO categories related to cell proliferation, including DNA replication, DNA recombination, cell division, cell cycle process, and nuclear division, were found to be overrepresented. This cluster also contained genes associated with organ development, such as multi-organism cellular process, cell wall organization, root development, and regulation of root meristem growth, indicating that cell division and proliferation are critical in generating callus. Furthermore, molecular function analysis identified microtubule binding, structural constituent of cytoskeleton, and auxin transmembrane transporter activity. This result supports that the cell cycle for callus expansion and proliferation was activated from day 4, and that this cluster could be an early stage for cell wall organization.

Cluster 3 comprised 3,053 genes, with highly expressed levels at day 7. The biological processes associated with this cluster were related to secondary metabolites (i.e., secondary metabolite biosynthetic process) and stress response (reactive oxygen species metabolic process, oxalate metabolic process, and cellular oxidant detoxification) as confirmed by GO analysis. Notably, this cluster contained genes involved in cell wall biogenesis, cellular polysaccharide metabolic process, lignin biosynthetic process, glucan metabolic process, and cell wall modification, suggesting active participation of genes in cell wall modification for callus formation at day 7. Additionally, KEGG pathway analysis revealed the presence of phenylpropanoid biosynthesis, starch and sucrose metabolism, glycine, serine and threonine metabolism, and biosynthesis of various plant secondary metabolites. Therefore, this finding suggests that the period leading up to day 7 played a crucial role in callus formation by triggering cell cycle activation and cell wall modification.

Cluster 4 consisted of 3,141 genes that exhibited a gradual increase until day 14, followed by rapid expression on day 28, and then a decrease in expression. GO terms related to stress response, such as response to toxic substances, cellular oxidant detoxification, reactive oxygen species metabolic process, and cellular oxidant detoxification, were identified in biological processes. KEGG pathway analysis revealed the presence of phenylpropanoid biosynthesis, MAPK signaling pathway, and plant-pathogen interaction. These findings suggest that genes involved in cell development are down-regulated, while stress-responsive genes are activated after day 14 during callus formation.

Clusters 5 and 6 exhibited gradually increasing expression patterns and contained 3,092 and 2,586 genes, respectively. Cluster 5 was associated with gene silencing by RNA, organophosphate biosynthetic process, carbohydrate derivative biosynthetic process, DNA modification, and DNA methylation or demethylation. Cluster 6 was associated with the recognition of pollen and cell, and pollen-pistil interaction. In contrast, clusters 7 and 8 demonstrated a sharp decline starting from day 1. Cluster 7 consisted of the highest number of genes (6,580). The GO terms associated with response to abscisic acid, regulation of the developmental process, negative regulation of gene expression, and negative regulation of biosynthetic process were identified in the biological process. On the other hand, cluster 8, consisting of 2,958 genes, exhibited a consistent decrease in expression levels starting from day 1. Overrepresented GO terms in this cluster included translational initiation, pyruvate metabolic process, response to cytokinin, and photosynthesis. Furthermore, the metabolic pathway analysis confirmed the involvement of carbon metabolism, glycolysis/gluconeogenesis, and carbon fixation in photosynthetic organisms. These findings suggest that the physiological mechanisms associated with callus formation are suppressed to halt unnecessary cellular events and conserve metabolic energy.

### 3.4 Transcriptome analysis of hormone-responsive gene expression

**Figure 4:**
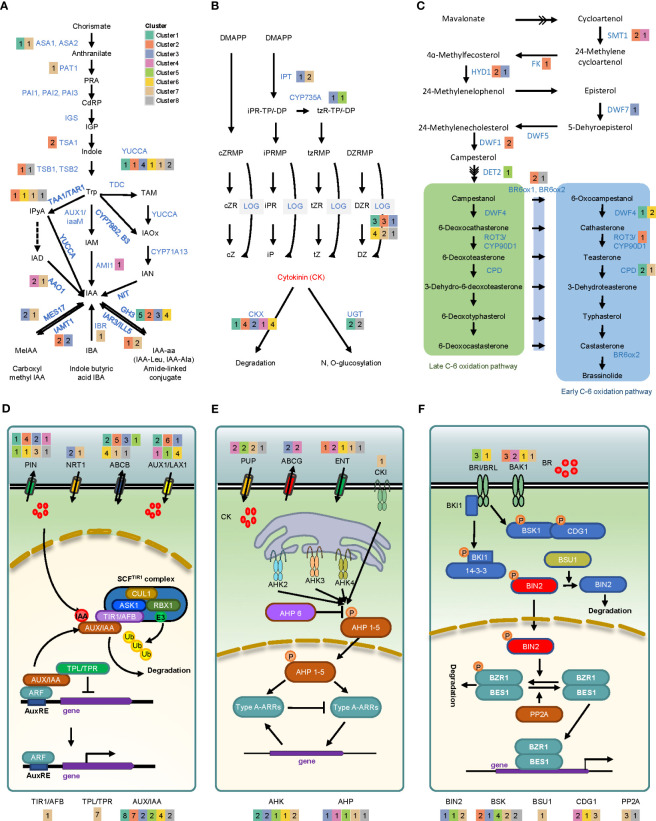
Gene expression profiling of phytohormone synthesis and signaling in auxin, cytokinin, and brassinosteroid. The upper pathways represent differentially expressed genes involved in the biosynthesis of auxin **(A)**, cytokinin **(B)**, and brassinosteroid **(C)**. The bottom pathways illustrate the differentially expressed genes associated with transporter and hormone signaling of auxin **(D)**, cytokinin **(E)**, and brassinosteroid **(F)**. The number in each colored box indicates the count of genes in each cluster. Key abbreviations include: ASA (anthranilate synthase alpha subunit 1), PAT1 (phosphoribosylanthranilate transferase 1), PAI (phosphoribosylanthranilate isomerase), IGS (indole glycerol phosphate synthase), TSA (tryptophan synthase alpha), TSB (tryptophan synthase beta), TAA1 (tryptophan aminotransferase 1), TAR1 (tryptophan aminotransferase-related protein 1), YUC (yucca), AAO1 (Arabidopsis aldehyde oxidase 1), AMI1 (amidase 1), NIT (nitrilases), GH3 (Gretchen Hagen 3), IAR3 (IAA-Ala resistant 3), ILL5 (IAA-Leu-resistant1-like 5), IBA (indole butyric acid), MES17 (methyl esterases 17), IAMT1 (IAA carboxyl methyltransferase 1), PIN (pin-formed), NRT1 (nitrate transporter 1), ABCB (ATP binding cassette subfamily B), AUX1 (auxin resistant1), LAX1 (like-auxin resistant1), CUL1 (cullin 1), ASK1 (*Arabidopsis* SKP1-like protein 1), RBX1 (RING-box 1), TIR1 (transport inhibitor response 1), AFB (auxin signaling F-box), IAA (indel-2-acetic acid inducible), TPL (tepless), TRR (TPL-related), IPT (isopentenyl transferase gene), LOG (Lonely guy), CKX (cytokinin oxidase/dehydrogenase), UGT (UDP-glycosyl transferase), PUP (purine permeases), ABCG (ATP-binding cassette transporter subfamily G), ENT (equilibrative nucleoside transporter), CKI (cytokinin independent), AHK (*Arabidopsis* histidine kinase), AHP (*Arabidopsis* histidine phosphotransfer protein), ARR (*Arabidopsis* response regulator), SMT (sterol methyltransferase), FK (fackel), HYD1 (hydra 1), DWF (dwarf), DET2 (de-etiolated), BR6OX (brassinosteroid-6-oxidase), ROT3 (rotundifolia 3), CPD (constitutive photomorphogenic dwarf), BRI (brassinosteroid-insensitive), BRL (brassinosteroid insensitive1-like), BAK1 (BRI1-associated receptor kinase 1), BKI1 (BRI1 kinase inhibitor 1), BSK1 (brassinosteroid-signaling kinase 1), CDG1 (constitutive differ- ential growth 1), BSU1 (BRI1 suppressor 1), BIN2 (brassinosteroid insensitive 2), BZR1 (brassinosteroid signaling positive regulator 1), BES1 (BRI1-EMS-suppressor 1), PP2A (protein phosphatase 2A).

We investigated the transcriptional changes of genes involved in biosynthesis, transport, and signaling of phytohormones to determine the effects of hormones during callus formation (**Table S6**). Auxin is synthesized from a central precursor, tryptophan (Trp), via three sub-pathways, including indole-3-acetaldehyde (IAM), indole-3-pyruvic acid (IPyA), and indole-3-acetonitrile (IAOX) (**Figures 4A, D**). Several key genes involved in the IPyA pathway were successfully identified. These genes encompass 4 tryptophan aminotransferase 2 (TAR2) and 18 YUCAA. Among the TAR2 genes, two (Glyma.04G186700 and Glyma.06G179000) were up-regulated. Additionally, up-regulation was observed in 2 YUC1/YUC4 genes, 3 YUC2/YUC6 genes, 1 YUC3/YUC7 gene, and 1 YUC5/YUC8/YUC9 gene. An alternative bypass route exists within the IPyA pathway for the synthesis of IAA, involving indole-3-acetaldehyde (IAD), and facilitated by aldehyde oxidase 1 (AAO1). We could confirm three orthologous genes of the AAO1 gene, with two genes (Glyma.02G272200 and Glyma.14G045100) being up-regulated over time. Additionally, we identified 2 amidase 1 (AMI1) and 3 CYP71A13 involved in IAM and IAOX pathways, respectively. One AMI1 (Glyma.20G232900) was up-regulated on day 28, but the expression value of CYP71A13 could not be determined. To maintain homeostasis in the level of free auxin, one approach is through IAA conjugation, which encompasses two processes: ester conjugation and amide conjugation. This mechanism is regulated by various developmental and environmental cues (Oüstin et al., 1998; Kowalczyk and Sandberg, 2001; Sanchez Carranza et al., 2016). We identified several genes related to the catabolism and transport of IAA, including Gretchen Hagen 3 (GH3), IAA-leucine resistant (ILR), ILR1-like (ILL), indole-3-butyric acid response (IBR), IAA carboxyl methyltransferase (IAMT), and MES17. GH3 and IAMT were mostly up-regulated, while IBR and MES17 were down-regulated. Furthermore, we discovered alterations in gene expression related to auxin transport. Specifically, genes such as 15 auxin inducible 1/auxin resistant 1 (AUX1/LAX1), 14 Pin-formed (PIN), 17 ABC subfamily b transporter (ABCB), and 3 nitroreductase 1 (NTR1) showed perturbations in their expression levels. We could also identify genes related to auxin signaling. Several genes associated with transport inhibitor response 1 (TIR1), which serves as an auxin receptor and interacts with Skp1/Cullin/F-box (SCF)-type E3 ubiquitin ligase complexes, exhibited a relatively down-regulated expression pattern. Furthermore, we observed a down-regulation of topless (TPL) and topless-related (TPR) genes, which function as inhibitors of auxin-responsive genes by interacting with auxin/indole-3-acetic acid (AUX/IAA) family. Conversely, during callus formation, we noted up-regulation in 23 AUX/IAAs, suggesting their active involvement in the process.

Cytokinin (CK) biosynthesis pathway involves enzymes such as IPT, CYP35A, and LOG, which utilize dimethylallyl diphosphate (DMAPP) as a precursor (Sakakibara, 2010; Frebort et al., 2011). In our study, we identified 10 orthologous genes belonging to the IPT family, namely IPT1, IPT2, IPT3, IPT5, and IPT9 (**Figures 4B, E**). Among these genes, only IPT3 (Glyma.03G151800) from cluster 3 exhibited up-regulation following callus induction. Among the genes involved in tZ biosynthesis, specifically CYP735A1/CYP735A2 genes, it was observed that two genes (Glyma.08G365000 and Glyma.18G297200) exhibited overall up-regulation. We identified 21 orthologous genes of LOG, including nine genes (LOG1 to LOG9) previously identified in *A. thaliana* (Tokunaga et al., 2012). Among these, 11 genes exhibited significant up-regulation. We discovered seven CK dehydrogenases/oxidases (CKX1~CKX7) involved in CK degradation for maintaining CK balance, along with 12 orthologous genes that were highly up-regulated. Additionally, we detected three types of proteins involved in CK transport: purine permease (PUP), equilibrative nucleoside transporter (ENT), and ATP-binding cassette (ABC) transporter (Bürkle et al., 2003; Chen et al., 2006; Ko et al., 2014), with 15, 9, and 4 genes identified, respectively. Among them, one ENT3 and two ENT8 proteins, which are involved in the uptake of iPR in yeast (Sun et al., 2005; Chen et al., 2006), were present in clusters 2 and 4, respectively. Additionally, two ABCG14 proteins belonging to cluster 3 were up-regulated, while PUP1, which facilitates CK uptake into the cell (Gillissen et al., 2000), exhibited down-regulation. Regarding CK signaling, 12 *Arabidopsis* histidine kinases (AHKs) and 12 *Arabidopsis* histidine phosphotransferase protein (AHPs) genes were identified. Among the AHKs, two AHK3 genes in cluster 7 were down-regulated, whereas 2 AHK4 genes in cluster 1 were highly up-regulated on day 1. In the case of AHPs, 3 AHP1 genes were up-regulated during callus formation, whereas other AHPs were either down-regulated or unexpressed.

Brassinosteroid (BR) is a steroidal phytohormone that plays a crucial role in cell division, elongation, and response to biotic and abiotic stress. The first compound of the brassinosteroid biosynthesis pathway, campesterol, is synthesized into brassinolide (BL) *via* campestanol-dependent and/or campestanol-independent pathways. In this study, we identified orthologous genes of sterol methyltransferase 1 (SMT1), fackel (FK), rotundifolia 3 (ROT3), and BR-6-oxiadase 1/2 (BR6OX1/BR6OX2) (**Figure 4C**). Among them, three SMT1s, one FK, one ROT3, and two BR6OX1/BR6OX2 were found to be up-regulated). Additionally, 14 biosynthesis pathway-related orthologous genes of the dwarf (DWF) family were confirmed. Among them, 2 DWF1s, 2 DWF3s (CPD), 3DWF4s, 1 DWF6 (DET2), and 1 DWF7 were found to be up-regulated. In BR signaling pathway, several key components, including brassinosteroid-insensitive (BRI1), BRI1-associated receptor kinase (BAK1), BRI1 suppressor (BSU1), brassinosteroid-insensitive 2 (BIN2), and serine/threonine protein phosphatase 2a (PP2A), were confirmed to be differentially regulated (**Figure 4F**). Among brassinosteroid receptor genes, two BRI1s showed no significant regulation, whereas BAK1s and BRI1-like genes (BRL1 and BRL2) were highly up-regulated. In the signaling pathway, two inhibitory BIN2s (Glyma.12G212000 and Glyma.13G289800) were up-regulated, whereas one BSU1 and four PP2As were down-regulated.

Furthermore, we observed the expressional dynamics of several genes involved in biosynthesis, transport, and signaling pathways of other phytohormones - ethylene, gibberellic acid, abscisic acid, and jasmonic acid - during callus induction. The expression patterns of these genes appeared to be perturbated, indicating the involvement of other hormone signals, in addition to auxin, CK, and BR, in the callus formation.

### 3.5 Analysis of transcriptional regulation related to cell cycle and cell wall development

**Figure 5:**
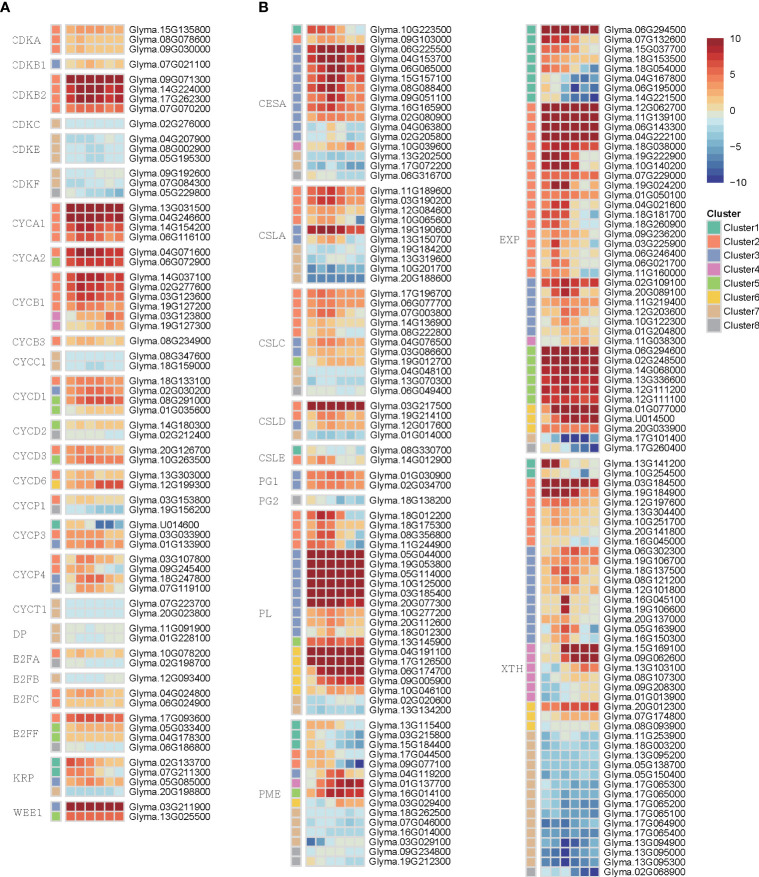
The heatmap represented differentially expressed genes involved in the cell cycle and cell wall during callus formation. **(A)** The heatmap shows the expression pattern of genes related to the cell cycle. **(B)** The heatmap displays the expression pattern change of genes related to the cell wall.

Because cell cycle and cell wall modification were known as key regulators during callus induction, we examined the transcriptional dynamics to gain insight into their role in callus formation (**Table S7**). We identified genes encoding cyclin (CYC), cyclin-dependent kinase (CDK), CDK inhibitor (CKI), retinoblastoma-related (RBR), E2F, and DP (Inzé and De Veylder, 2006; Qi and Zhang, 2020; Shimotohno et al., 2021) (**Figure 5A**). CYC genes in plants are encoded by a multi-gene superfamily, which is categorized into seven classes: A, B, C, D, H, P, and T. In our analysis, we identified 7, 8, 2, 14, 2, 11, and 3 orthologous genes for each respective class. Among them, several genes encoding D-type, A-type, and B-type CYCs play a role in the G1-to-S, S-to-M, and G2-to-M transition, respectively. Interestingly, these genes exhibited higher expression levels during callus formation compared to other members of the CYC family in our study. The CDK gene, known to interact with CYC genes, is classified into 11 families designated from A to K. Among 21 orthologous CDK genes, CDKB genes responsible for facilitating the transition from G2 to M phase of the cell cycle through interaction with CYCA, CYCB, or CYCD displayed notably high expression levels. Taken together, these findings indicate that G2-to-M phase, which is the process that drives cell division in the cell cycle, is dynamically activated during callus formation. Kip-related protein (KRP) and WEE1, which are included in the CKI family, act as negative regulators in the G1/S transition and G2/M transition, respectively. Contrary to our expectations, these genes were up-regulated during callus formation. This phenomenon may be attributed to the dynamic expression patterns of cell cycle-related genes, which exhibit changes that occur at a faster rate than observable time intervals between samples. Additionally, the samples used in the study were derived from a mixture of different callus cell types, contributing to the observed variability in gene expression. Therefore, the highly expressed pattern of CKI genes may indicate that the cell cycle is dynamically activated by these genes.

To uncover the transcription regulation in the cell wall coupled with the cell cycle, we investigated several genes involved in cell wall synthesis and modification (**Figure 5B**). The cellulose synthase (CesA) and cellulose synthase-like protein (CLS) are involved in cell wall synthesis. We validated that 13 CesA and 18 CLS genes exhibited high expression levels during callus formation. Furthermore, we discovered various genes associated with cell wall modification, such as pectin methylesterase (PME), xyloglucan endotransglycosylase/hydrolase (XTH), polygalacturonase (PG), expansin (EXP), and pectin lyases (PL). This observation suggests that these genes undergo substantial regulation during callus formation, indicating that wounding stimuli influence the process of cell wall synthesis and modification.

### 3.6 Identification of transcription factors and associated genes in relation to callus formation

**Figure 6:**
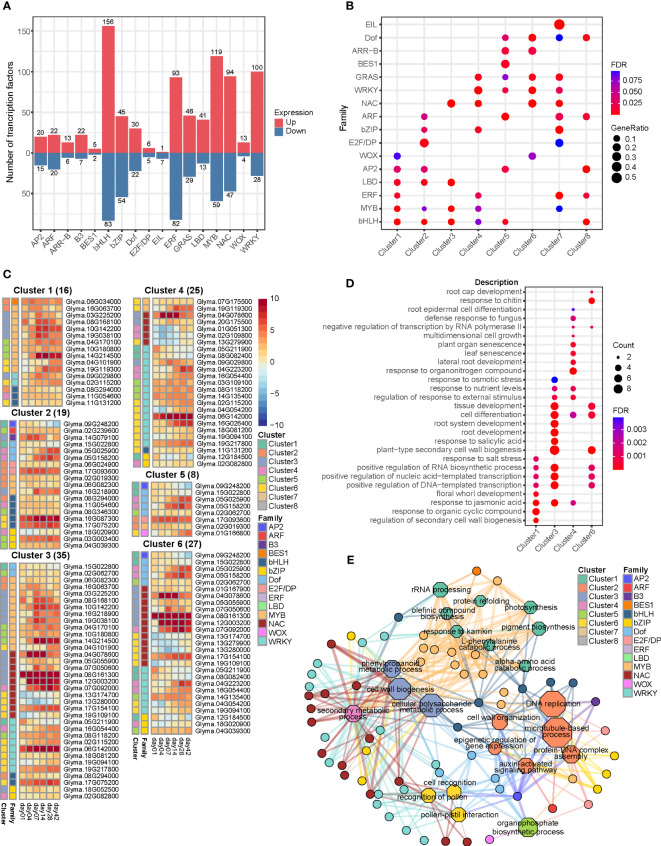
Analysis of transcription factors differentially expressed during callus formation. **(A)** Bar plot displaying the number of differentially expressed genes associated with transcription factors. **(B)** Dot plot demonstrating the result of the enrichment analysis of transcription factors using a hypergeometric distribution, filtered with a false discovery rate (FDR) threshold of < 0.1. **(C)** Heatmap depicting the expression patterns of genes encoding differentially expressed transcriptional regulators, identified through TF enrichment analysis in PlantRegMap. These transcription factors govern the transcriptional dynamics of differentially expressed genes within each cluster. **(D)** Dot plot presenting the result of the GO enrichment analysis for the enriched transcription factors. **(E)** Network diagram illustrating the interaction between the enriched transcription factors and the GO terms associated with their target genes.

In order to explore the transcriptional regulation changes during callus formation, we obtained a comprehensive set of 3,866 TFs. Among them, we identified 1,309 differentially expressed TFs belonging to 17 distinct TF families (**Table S8**). The largest TF families among these genes were the basic/helix loop helix (bHLH), comprising 239 genes, and the ethylene responsive factor (ERF) family, which consisted of 175 genes. These families exhibited significant changes in gene expression following induction. Other TF families also exhibited significant expression changes, including 178 MYBs, 141 NACs, 128 WRKYs, and 99 bZIPs (**Figure 6A**). To explore the relationship between clusters and TF families, we conducted an enrichment analysis using hypergeometric distribution in hyperR^8^. This analysis involved assessing the overrepresentation of TF families within each cluster (**Figure 6B**).

Out of the 175 genes implicated in ERF transcription factor, 53% (93 genes) were up-regulated during callus formation and plant regeneration. In addition, the expression of WIND transcriptional regulators, which are known to play a role in callus initiation under wounding stress, was also altered after induction (Ikeuchi et al., 2017). Specifically, two WIND1 genes (Glyma.06G105000 and Glyma.13G088100) and two WIND4 genes (Glyma.05G179900 and Glyma.08G137600) were identified and grouped into clusters 6 and 1, respectively. Other genes, such as estrogen receptor 1/2 (ESR1/ESR2; Glyma.02G039300), ERF114 (Glyma.04G201900, Glyma.05G200100, and Glyma.06G163700), and ERF115 (Glyma.08G257300), were also found to be differentially expressed and grouped into cluster 1. On the contrary, a total of 71 genes exhibited down-regulation, including dehydration-responsive element-binding (DREB), abscisic acid insensitive 4 (ABI4), and ethylene responsive element binding protein (EBP). Among the induced TFs, 35 apetala2s (AP2s) displayed altered expression and were over-represented in clusters 1, 2, 5, and 8. Aintegumenta-like (AIL) genes of AP2 family, which are expressed in dividing tissues and play a pivotal role in the developmental process, were up-regulated following callus induction (Kareem et al., 2015; Ikeuchi et al., 2017; Shen et al., 2020). During callus formation, orthologous genes of plethora1 (PLT1), PLT2, and aintegumenta (ANT) were up-regulated, while PLT3, PLT5, and PLT7 exhibited down-regulation.

We observed that 17 wuschel-related homeoboxs (WOXs) involved in cell division and proliferation were differentially expressed and over-represented in clusters 1 and 6. Notably, WOX1 (Glyma.09G177800), WOX5/WOX7 (Glyma.02G254800, Glyma.11G227800, and Glyma.18G029700), and WOX4 (Glyma.04G040900, Glyma.06G041800, Glyma.14G084600, and Glyma.17G240300) were highly up-regulated during callus initiation. WOX11/WOX12 (Glyma.03G007600 and Glyma.19G118400), known as the activator of WOX5/WOX7 in root primordia initiation (Hu and Xu, 2016), were down-regulated during callus formation. In parallel, a total of 54 LBDs involved in callus initiation and plant regeneration were highly expressed and over-represented in clusters 1, 2, and 3. Among them, LBD16 (Glyma.03G161400, Glyma.10G035000, Glyma.13G121300, and Glyma.19G162900), LBD18 (Glyma.01G143900, Glyma.03G023000, Glyma.09G268700, and Glyma.18G221000), and LBD17/LBD29 (Glyma.03G161500, Glyma.10G035100, Glyma.13G121400, and Glyma.19G163000), related to the downstream of ARF7 and ARF19 in callus formation (Okushima et al., 2007), were up-regulated.

A total of 42 ARF, 19 ARR, 7 BES1 (BRI1-EMS suppressor 1), and 8 EIL (ethylene insensitive3-like) TFs, which are associated with hormone signaling, were identified. Among the ARFs related to auxin signaling, the orthologous genes of ARF3, ARF4, and ARF5, which activate the repressor of shootmeristemless (STM) for enabling the differentiation of cells (Chung et al., 2019), were up-regulated. In addition, ARF10 and ARF16, which activate the callus initiation to repress the expression of ARR15 (Liu et al., 2016), also were up-regulated. In contrast, ARF7/ARF19 (Glyma.13G112600 and Glyma.17G047100), known as a key regulator during callus formation, were down-regulated. The 19 ARR TFs related to cytokinin signaling were over-represented in clusters 5 and 6. During callus formation, ARR1 and ARR12 promote the process by activating cell cycle reentry through the regulation of CYCD3;1 influenced by wounding stimuli (Ikeuchi et al., 2013; Ikeuchi et al., 2017). We found that 1 ARR1/ARR2, 3 ARR11s, and 6 ARR12s were up-regulated during callus formation. The BES1 is related to brassinosteroid signaling. Our study unveiled that a mere 7 genes exhibited significant differential expression, and enrichment analysis demonstrated over-representation in cluster 5. In addition, 3 orthologous genes of BES1 (Glyma.06G034000, Glyma.14G076900, and Glyma.17G248900) were highly expressed during callus formation. Regarding EIL transcription factors involved in ethylene signaling, the majority (7 out of 8) of the genes exhibited down-regulation, indicating their over-representation in cluster 7. Overall, these results indicate that TFs responsive to wounding and hormones were dynamically changed during callus formation.

To ascertain TFs responsible for regulating the transcriptional dynamics of differentially expressed genes within each cluster during callus formation, we performed TF enrichment analysis using genes from each cluster in the PlantRegMap database. Furthermore, we conducted GO enrichment analysis to explore the enriched TFs and their target genes. Additionally, we employed gene regulatory network analysis to unravel the biological significance of these findings (**Figures 6C–E**). A total of 67 TFs were identified under the following conditions: p-value < 0.05 and inclusion in clusters 1-6. Cluster 1 was governed by 16 TFs associated with biological processes such as secondary cell wall biogenesis, organic cyclic compound, and regulation of DNA-templated transcription. On the other hand, cluster 2, regulated by 19 TFs, did not exhibited GO terms. However, we verified that ARF TFs and B3 TFs, specifically present in clusters 5 and 3, respectively, exclusively targeted this cluster. Cluster 3 was governed by 35 TFs associated with GO terms related to callus development, including secondary cell wall biogenesis, root development, cell differentiation, and tissue development. In contrast, cluster 4, controlled by 25 TFs, is involved in senescence-related processes such as leaf senescence and plant organ senescence. Cluster 5 consisted of 8 TFs, including 4 Dofs, 1 AP2, 1 E2F/DP, 1 MYB, and 1 WOX. Notably, WOX TF specifically targeted cluster 5 and was not found in any other clusters. On the other hand, cluster 6 was regulated by 27 TFs and exhibited a high representation of GO terms related to cell development, similar to cluster 3.

Our gene regulatory network analysis uncovered the specific biological processes in each cluster that were targeted by enriched TFs. Out of 27 enriched NAC TFs, we discovered the orthologous gene (Glyma.17G154100) corresponding to ANAC011/ANAC071/ANAC096. This key regulator, known to respond to wounding signals, governs the expression of 126 genes in cluster 3 and 119 genes in cluster 6. AP2 (Glyma.09G248200), an orthologous gene of BBM, was found within cluster 1 and exerted regulatory control over 1299, 1043, and 858 genes in clusters 2, 5, and 6, respectively. These genes are associated with auxin-activated signaling pathway, cell wall organization, cell cycle process, and cell division. ARF8 (Glyma.02G239600) governs the regulation of 54 genes within cluster 2, encompassing processes such as auxin-activated signaling pathway, microtubule-based process, and cytoskeleton organization. BES1 (Glyma.06G034000) exhibits significant up-regulation during callus formation and controls the regulation of 121 genes within cluster 1. These genes are involved in various processes such as photosynthesis, light harvesting in photosystem I, rRNA processing, and rRNA metabolic process. OBF-interacting protein 1 (OBP1; Glyma.02G062700), a member of the DOF family, is present in cluster 5 and exerts regulatory control over clusters 3, 5, and 6. It governs genes associated with cell wall biogenesis and cell cycle process. E2F/DP (Glyma.06G024900 and Glyma.17G093600), which plays a role in the cell cycle, is present in cluster 2 and regulates clusters 2 and 5. Additionally, B3 (Glyma.14G079100) exhibits high expression and governs the regulation of 133 genes within cluster 2. Overall, these transcription factors control various biological processes such as DNA replication, cell cycle, nucleosome assembly, and cell division. WUSCHEL (WUS; Glyma.01G166800) governs the regulation of 89 genes in cluster 5, which are involved in processes such as organophosphate biosynthesis, nucleoside phosphate metabolism, and carbohydrate derivative biosynthesis.

### 3.7 Analysis of gene expression changes during the initial 7 days of callus formation

**Figure 7:**
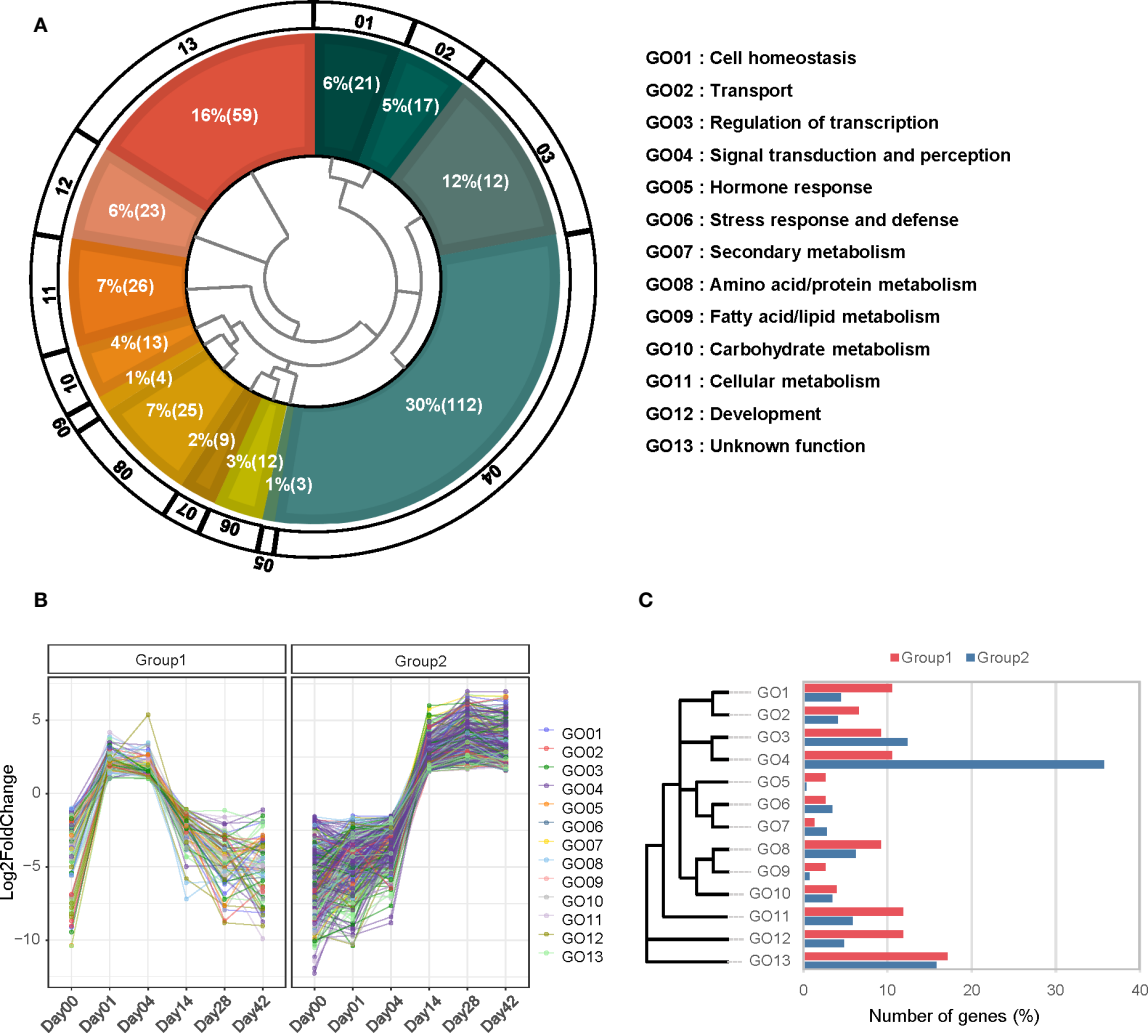
GO categorization of differentially expressed genes expressed at 7 days after wounding (7DAW). **(A)** The 415 differentially expressed genes (DEGs) are divided into two groups based on their expression at 7DAW. The inner space presents a hierarchy of 15 GO categories, with the percentage and number of genes included in each category indicated by white letters. **(B)** Expression patterns of group 1 (left plot) and group 2 (right plot) are shown. Each group is filtered based on the specified criteria mentioned below. **(C)** The percentage of genes associated with each GO category is separated into two groups. The hierarchical diagram for each GO category is displayed on the left side. Genes belonging to group 1 are represented in blue, while genes belonging to group 2 are shown in orange.

To explore the phenomenon that the day 7 acted as the boundary point of callus morphology from cell division and growth to senescence (**Figures 1A**g,h, **2A**), we analyzed the expression pattern comparing each sample to day 7 and filtering with the following condition: the first group (log2FC < -1 at 0 DAW, log2FC > 1 from day 0 to 4, and log2FC < -1 from day 14 to 42) and the second group (log2FC < -1.5 from day 0 to 7 and log2FC > 1.5 from day 7 to 42) (**Figure 7B**). We identified 76 and 291 genes involved in the two groups, respectively. In order to perform a more detailed analysis of these patterns, we took 13 representative categories from the gene ontology and manually classified them into two groups (**Figures 7A, C**; **Table S9**).

Group 1 was highly related to cell homeostasis (GO 01), hormone response (GO 05), cellular metabolism (GO 11), and development (GO 12), including genes related to cell multiplication and enlargement similar to cell growth such as deeper rooting 1-like (DRO1-like), CYCA2;1, WOX5, and EXP. The early stages of callus development seem to involve an increase in cell size during the log phase. EXP (Glyma.19G222900) was expressed in response to wounding and increases cell size by loosening the cell wall (Cho and Cosgrove, 2000). When callus is generated in auxin-rich conditions, it follows a root developmental program (Ikeuchi et al., 2017). One of the representative genes for root formation, DRO1-like (Glyma.07g040800), was highly expressed and contributes to root meristem development, leading to the organized expression of regulators of root tissue (Grimplet et al., 2017; Ikeuchi et al., 2017). In response to hormones, particularly auxin, small auxin up-regulated RNA (SAUR; Glyma.02G049200), type I inositol polyphosphate 5-phosphatase 4 (IP5P4; Glyma.02G145600), and naked pins in YUC mutants 4 (NPY4; Glyma.17G161500) were up-regulated in the early stages and played a role in development (**Figure 7B**). Contrary to group 1, group 2 was over-represented genes involved in signal perception and transduction (GO 04), including stress-responsive factors such as leucine-rich receptor-like kinase (LRR), cysteine-rich receptor-like protein kinase (CRR), and G-type lectin S-receptor-like serine/threonine-protein kinase (GsSRK). These genes are mainly stimulated by stress-responsive phytohormones such as ABA and ethylene (Sun et al., 2017). These results suggest that the genes involved in root formation, cell wall expansion, and auxin response were highly regulated for callus initiation and proliferation during the initial 7 days, whereas stress response was highly activated after day 7.

## 4 Discussion

**Figure 8:**
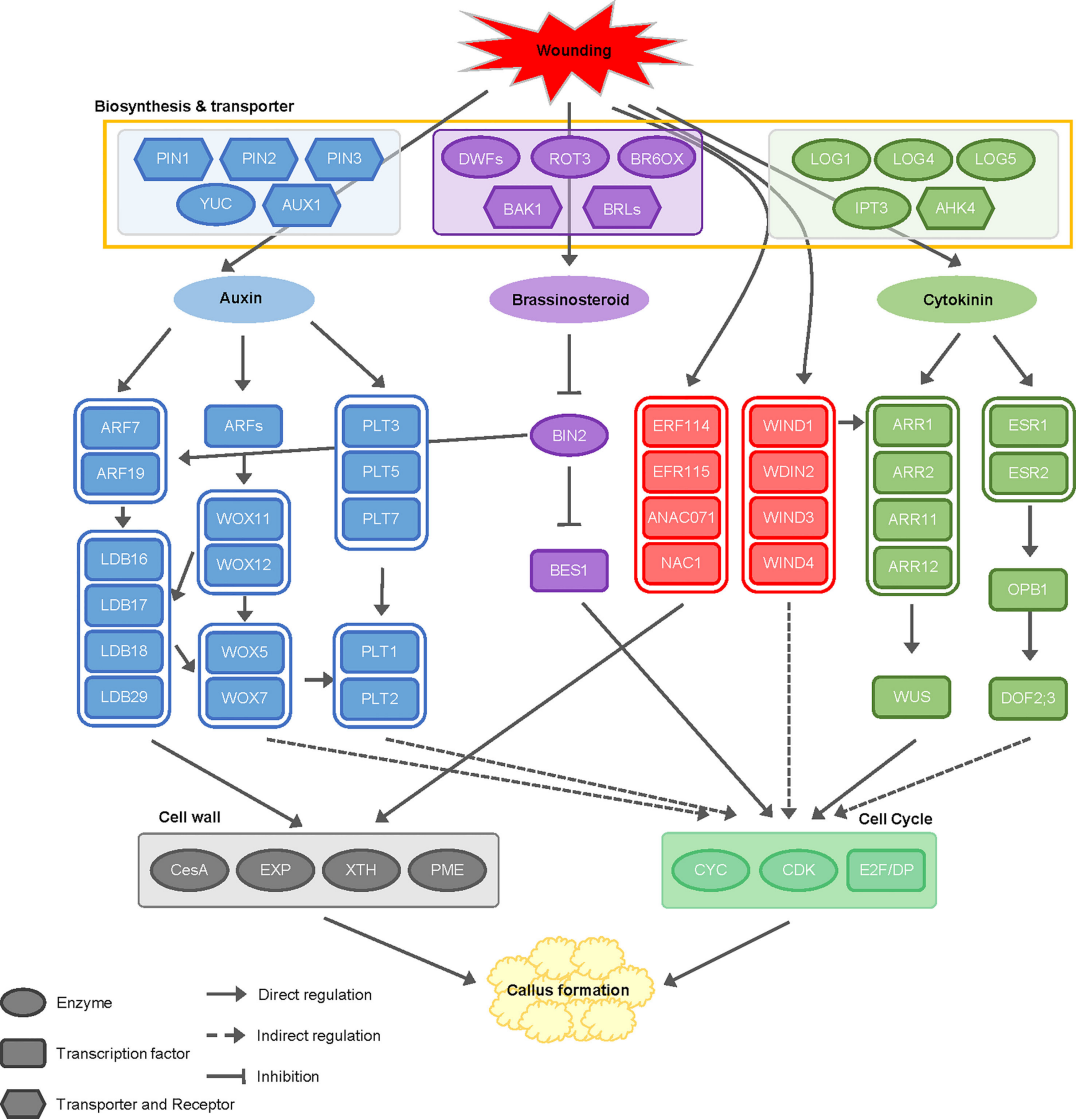
Schematic diagram of cellular mechanisms and interactions between wounding and hormones during callus formation. This diagram illustrates the cellular mechanism underlying the interactions between wounding signals and phytohormones, including auxin, cytokinin, and brassinosteroid, during callus formation. The regulatory genes involved in these processes are color-coded: genes regulated by wounding signals are indicated in red, genes related to auxin are shown in blue, genes activated by cytokinin signaling are depicted in green, and genes involved in brassinosteroid signaling are represented in purple. PIN (pin-formed), YUC (YUCCA), AUX (auxin transporter protein 1), ARF (auxin response factor), LBD (lateral organ boundaries domain), WOX (wuschel-like homeobox), PLT (plethora), BRI1 (brassinosteroid insensitive 1), BIN2 (brassinosteroid insensitive 2), BES1 (BRI1-EMS-suppressor 1), ERF (ethylene response factor), ANAC071 (NAC domain-containing protein 71), NAC1 (Nucleus accumbens-associated 1), WIND (Wound induced dedifferentiation), LOG (lonely guy), IPT (isopentenyl transferase gene), AHK (*Arabidopsis* Histidine Kinase), ARR (*Arabidopsis* response regulator), WUS (wuschel), ESR (enhancer of shoot regeneration), OPB1 (OBF binding protein 1), DOF2;3 (DNA binding with one finger 2;3).

Studies on callus formation have described two separate mechanisms: the wounding-induced pathway and the CIM-induced pathway (Ikeuchi et al., 2017). However, since all biological processes in plants are interconnected rather than being separate, the pathways related to callus formation are expected to be interlocked as well. When responding to wounding stress, cell reprogramming is activated rapidly and strongly to generate callus, and then the balance between two phytohormones, auxin and cytokinin, determines cell fate (Iwase et al., 2018). Since callus is induced using explants from half-seed or cotyledon in soybean, wounding stress is inevitable (Sairam et al., 2003; Thibaud-Nissen et al., 2003; Jiang et al., 2010). Therefore, it is difficult to divide the callus formation mechanism by wounding and hormones precisely. In this regard, we performed transcriptome analysis on soybean callus induced by wounding and hormones to understand how they affect callus formation. Our data revealed that the complex interaction of pathways by wounding and external hormones activates callus formation (**Figure 8**).

### 4.1 Recognizing the temporal dynamics: The distinct stages of callus formation in *G. max*

Based on the analysis of time-series DEGs, which compared each sample to the control (day 0), our research confirmed that 50.31% of all genes revealed transcriptional dynamics at least once during callus formation. Plants tend to activate various metabolic pathways to resist abiotic stress, such as pathogen attack, wounding, UV exposure, high light, and nutrient deficiencies (Akula and Ravishankar, 2011; Yoon et al., 2020). In our study, wounding-treated cotyledon was incubated under abnormal conditions, such as dark and auxin-rich, to induce callus. These processes are necessary for our research to uncover the transcriptional dynamic during callus formation in soybean. So, stress-related TFs such as ERF, MAC, MYB, MYC, and WRKY were highly expressed, as well as hormonal pathways (i.e., auxin, CK, and BR), cell walls, and cell cycles.

Our clustering analysis with DEGs identified genes that change dynamically over time, so we classified dramatic alterations in the expression pattern of genes related to callus formation. Clusters were divided into three classes according to the transcriptional dynamics during callus formation, as shown in **Figure 3A**: ectopic regulation (clusters 1, 2, 3, and 4), positive regulation (clusters 5 and 6), and negative regulation (clusters 7 and 8). The ectopic regulation class suggests that transcriptional dynamics of the genes involved in specific time points (i.e., days 1, 4, 7, and 28) affect callus formation by responding to hormones and wounding stress. Among them, many genes encoding enzymes for auxin signal transduction and cell proliferation were involved in clusters 1 and 2, which showed to be associated with callus induction. Also, genes related to cell wall modification, which are key regulators in callus development, were differentially expressed before and after day 7, including in cluster 3. In other words, the auxin pathway and cell division for callus induction are initially activated, and cell wall modification, in turn, is rapidly activated for callus proliferation. In contrast, the negative regulation class showed that the genes related to physiological mechanisms are repressed, suggesting that dynamic changes of many cellular events are essential for callus formation, and physiological mechanisms in the normal life cycle are shut down to save the metabolic energy for an abnormal cellular phenomenon.

### 4.2 Influence of endogenous and exogenous hormones on callus formation

For several decades, a multitude of studies have consistently demonstrated that callus development is significantly affected by various phytohormones, notably auxin and cytokinin, which play a pivotal role in triggering the callus formation (Ikeuchi et al., 2013; Ikeuchi et al., 2017; Lee and Seo, 2017; Ikeuchi et al., 2019). Based on our findings, we suggest that the modulation of gene expression during callus formation is governed by the interplay between auxin, cytokinin, and brassinosteroid, working in conjunction with wound signaling mechanisms (**Figure 8**).

The findings of this study provide confirmation that genes associated with IAM and IPyA were observed to be up-regulated, suggesting the activation of the auxin biosynthesis pathway as a response to wounding. Furthermore, a majority of genes involved in transporter activity exhibited up-regulation, indicating the enhanced expression of genes responsible for auxin transporters, facilitating the influx of external auxin into the cellular environment. Moreover, genes associated with auxin degradation, specifically GH3 and IAMT1, were observed to be up-regulated (**Figure 4A**). Based on these findings, it appears highly probable that the level of auxin increased as a result of auxin biosynthesis activation and enhanced transporter activity induced by both wounding and external auxin. Consequently, this leads to the degradation of auxin to uphold auxin homeostasis within the cell.

CK, a crucial hormone along with auxin in callus formation, is synthesized by three biosynthesis-related genes (i.e., IPT, CYP735A, and LOG) in *Arabidopsis*. In IPT, which is responsible for the initial step in the cytokinin biosynthesis pathway, AtIPT5 and AtIPT7 were induced by auxin, whereas AtIPT1, AtIPT3, AtIPT5, and AtIPT7 were suppressed by CK in shoot meristem (Cheng et al., 2012). In this study, we identified the orthologous genes in soybean, of which only IPT3 exhibited up-regulation (**Figure 4B**). Based on our analysis, we deduce that IPT3 is closely associated with the CK response triggered by externally applied CK during callus formation. The up-regulation of CYP73As and LOGs, which play a role in the subsequent step, provides evidence for the activation of CK biosynthesis pathway during callus formation. Within the context of CK signaling, the expression profiles of genes encoding AHK, AHP, ARR, and ESR were observed to be up-regulated. This up-regulation subsequently leads to the activation of CYCD, a key regulator of the cell cycle. These findings strongly suggest that CK synthesis is triggered by wounding signals and, in turn, influences callus formation by promoting cell cycle progression through intricate interactions with signaling factors.

Despite active research, the precise contribution of BR, a phytohormone crucial for development and growth, to callus formation remains uncertain. However, in this study, a significant portion of genes related to BR biosynthesis exhibited up-regulation, shedding light on their potential involvement in the process. Notably, DWF4, a gene already recognized for its robust expression in dividing callus cells (Chung et al., 2011), exhibited an exceptionally elevated level of expression. Moreover, BAK1 and BRLs, which are recognized for their role in regulating various developmental processes, such as cell division, elongation, root growth, vascular differentiation, and resistance to stressors (Oh et al., 2020), exhibited significant up-regulation. This observation provides compelling evidence that the accumulation of BR within cells triggers the activation of the cell division process, ultimately leading to callus formation (**Figure 4C**). Several studies have explored the signaling pathway, uncovering the role of BIN2 as a positive regulator through the BIN2-ARF-LBD complex, while also noting the activation of BES1/BZR1 during cell division (Wei and Li, 2016; Rane et al., 2017). Consistent with these findings, our research identified distinct expression patterns of these genes throughout callus formation, providing further evidence that injury-related stimuli trigger the synthesis of BR. Consequently, this leads to the stimulation of cell division as a consequential outcome.

### 4.3 Dynamic regulation of cell wall and cell cycle mechanism during callus formation

Several studies have examined transcriptional regulators that play a role in the initiation and progression of callus, such as ARF, LBD, WOX, WIND, and AR2/ERF in different species (Ikeuchi et al., 2013; Ikeuchi et al., 2017; Du et al., 2019; Suo et al., 2021). ERF114, ERF115, ANAC071, and ANAC096 are activated in organ development and regenerative responses *via* cell wall damage by wounding (Ikeuchi et al., 2017; Zhang et al., 2022). Moreover, ANAC071 is known to directly regulate XTH19 and XTH20, which promote pith cell proliferation (Pitaksaringkarn et al., 2014). The data we obtained demonstrated a substantial up-regulation in the expression pattern of these genes, thereby corroborating previous findings. Consequently, our results strongly indicate that wounding stimuli play a significant regulatory role in cell wall modification during callus formation, mediated by wounding-responsive transcription factors.

Wounding-Induced Networked Developmental Regulator 1 (WIND1), which has homologs WIND2, WIND3, and WIND4, is involved in callus formation and cell division in the apical meristem and is induced by wounding (Iwase et al., 2011). Under wound stress, WIND1 has a direct regulatory effect on ARR1 and ARR12, which participate in cytokinin signaling, as well as on ESR1 and ESR2, which were involved in shoot meristem development (Ikeuchi et al., 2017). In our study, we confirmed that WIND1 and WIND 4 were differentially expressed at day 1, and ARR1/ARR2, ARR11, ARR12, and ESR1/ESR2 exhibited an increase in the expression profile. Furthermore, ESR2 regulates OBP1, which is the DOF transcriptional regulator. OBP1 plays a role in cell division by targeting the core cell cycle regulator CYCB3;3 and the S phase-specific transcription factor DOF2;3 (Skirycz et al., 2008; Ikeuchi et al., 2013). WUS, a well-known marker gene associated with shoot meristem development, also plays a role in callus formation by governing the fate of meristem cells (Ikeuchi et al., 2013). Analysis of the RNA-seq data unveiled an upsurge in the expression levels of OBP1, DOF2;3, CYCB3;3, and WUS genes. Notably, OBP1 (Glyma.02G062700) and WUS (Glyma.01G166800) exerted significant influence on the transcriptional dynamics of genes within the ectopic regulation cluster. These findings strongly suggest that the cytokinin-dependent pathway involved in shoot meristem development is activated in response to wound stress, potentially playing a role in callus formation by modulating the fate of meristematic cells.

ARFs, TFs that are under the regulation of auxin, have been the subject of extensive research due to their pivotal role in plant development and callus formation (Chen et al., 2011; Li et al., 2016). ARF7 and ARF19 have been recognized as key players in the process of lateral root development by facilitating the activation of downstream transcription factors, including LBD16, LBD17, LBD18, and LBD29, whose involvement in this pathway is well-established (Okushima et al., 2007). Additionally, in the initiation stage of root primordia, the expression of LBD genes was governed by WOX11 and WOX12 (Hu and Xu, 2016). Moreover, it has been observed that LBD18 directly promotes the emergence of lateral roots through the regulation of EXP14 (Lee et al., 2013). Conversely, LBD16 plays a role in root regeneration and lateral root formation, directly activating FAD-BD. This activation occurs through the formation of a heterodimeric complex with bZIP59, thereby facilitating cell wall remodeling (Goh et al., 2012; Liu et al., 2018; Xu et al., 2018). In contrast, LBD29 has been found to regulate PME2 during the process of cell wall modification in callus formation (Xu et al., 2018). Our investigation corroborated the up-regulation of LBD and its downstream genes, particularly those implicated in cell wall modification. However, intriguingly, we observed a down-regulation of upstream genes, including ARF7 and ARF19. According to previous research in *A. thaliana* (Okushima et al., 2007), it was anticipated that the ARF7 and ARF19 genes would have already exhibited expression and initiated the activation of downstream genes prior to 1-day time point examined in our study. Therefore, the noteworthy up-regulation of LBD and its downstream genes observed during callus formation aligns perfectly with this expectation. Consequently, the expression of ARF TFs is induced by auxins, subsequently promoting the expression of LBD genes and facilitating the activation of cell wall modification processes.

WOX5 assumes a critical role as a transcriptional regulator in both lateral root development and callus formation. Its regulation is mediated by LBDs, WOX11, and WOX12, while it acts as a regulator of PLT1 and PLT2 (Ding and Friml, 2010; Sugimoto et al., 2010; Hu and Xu, 2016). Moreover, WOX7 and WOX14, which interact with WOX5, are recognized for their pivotal roles in pluripotency acquisition during callus formation, as well as in lateral root development and vascular cell differentiation (Kong et al., 2016; Denis et al., 2017). Our investigation made a noteworthy observation of substantial up-regulation of WOX5 and WOX7 over a period of seven days. However, it is important to note that orthologous genes of WOX11 and WOX12 were not identified in *G. max*. Interestingly, PLT3, PLT5, and PLT7 were identified as key regulators of root development, playing a crucial role in *de novo* shoot regeneration and the acquisition of cell pluripotency through the regulation of PLT1 and PLT2 activity (Kareem et al., 2015; Bustillo-Avendaño et al., 2018). Furthermore, another study reported an up-regulation of PLT family genes in *A. thaliana* as early as one hour after wounding, which triggers callus formation (Ikeuchi et al., 2017). Through our research, we noted a decline in the expression levels of PLT3, PLT5, and PLT7 genes starting from day 0, while the expression of PLT1 and PLT2 genes showed an increase. These findings indicate that our RNA-Seq analysis was conducted after day 1, preventing us from confirming the rapid up-regulation of PLT3, PLT5, and PLT7 within one hour. However, we were able to observe the expression of downstream genes, such as PLT1 and PLT2. These results imply that WOX5 plays a critical role in regulating PLT1 and PLT2, and the interplay between auxin and wounding stimulates the mechanism of root meristem development. Ultimately, this contributes to callus formation by influencing meristematic fate.

## 5 Conclusion

In this study, our objective was to explore the transcriptional regulation of callus formation in soybean under the influence of wounding signals and phytohormones. To gain deeper insights into the intricate mechanisms involved in this process, we performed a thorough transcriptome analysis of *G. max*. Employing a time-series gene expression profiling approach, we successfully identified key genes associated with transcription factors, biosynthesis, transporters, and signaling pathways related to phytohormones. These findings collectively contribute to a comprehensive understanding of the underlying mechanisms governing callus formation. Notably, this study goes beyond elucidating individual components and explores the intricate interactions among major phytohormones involved in callus formation, including auxin and cytokinin (as previously explained), and the newly implicated brassinosteroid. Such a broader investigation will enhance our understanding of this complex phenomenon. Our results indicate that the coordinated interplay of wounding, auxin, cytokinin, and brassinosteroid signaling pathways triggers the activation of genes involved in determining the fate of meristematic cells. This, in turn, initiates processes such as cell wall modification and cell cycle reentry, ultimately leading to the formation of callus. By unveiling these intricate interactions, our study sheds light on the complex regulatory network orchestrating callus formation in soybean.

## Data availability statement

The datasets presented in this study can be found in online repositories. The names of the repository/repositories and accession number(s) can be found below: https://www.ncbi.nlm.nih.gov/bioproject/PRJNA949161, PRJNA949161.

## Author contributions

J-SP: conceptualization, data curation, formal analysis, Writing-original draft. YC: conceptualization, investigation, formal analysis, experiment, writing – original draft, M-GJ: data curation, validation, Y-IJ: data curation; validation, J-HH: data curation, validation, H-KC: conceptualization, funding acquisition, Supervision, Project administration, writing – original draft, writing – review & editing. All authors contributed to the article and approved the submitted version.

## Footnotes

1
http://daehwankimlab.github.io/hisat2

2
https://htseq.readthedocs.io/en/master

3
http://www.interactivenn.net/

4
https://phytozome-next.jgi.doe.gov

5
http://plantregmap.gao-lab.org

6
http://planttfdb.gao-lab.org

7
https://tgil.donga.ac.kr

8
https://bioconductor.org/packages/release/bioc/html/hypeR.html

## Supplementary material

Supplementary Figure 1 Summary of similarity among samples. The enrichment maps show the top 20 enriched biological processes based on the analysis of top 1000 genes in PC1 **(A)** and PC2 **(B)**, respectively.

Supplementary Figure 2 Confirmation of RNA sequencing results using real-time PCR analysis. The expression values obtained from RNA-seq were validated by real-time PCR analysis. The left and right scales represent RNA-Seq and qRT-PCR values, respectively. Error bars indicate the standard error calculated from three biological replicates and three technical replicates. ERF15 (Glyma.01G206600); AOS1 (Glyma.04G035000); CYCA2;1 (Glyma.04G071600); ARF19 (Glyma.09G072200); LBD15 (Glyma.03G161500); LOX3 (Glyma.03G264300); OBP4 (Glyma.05G018100); NAC34 (Glyma.08G173400); WOX5 (Glyma.02G254800).

## References

[B1] Akula R. Ravishankar G. A. (2011). Influence of abiotic stress signals on secondary metabolites in plants. Plant Signal. Behav. 6, 1720–1731. doi: 10.4161/psb.6.11.17613 22041989 PMC3329344

[B2] Anders S. Pyl P. T. Huber W. (2015). HTSeq–a Python framework to work with high-throughput sequencing data. Bioinformatics 31, 166–169. doi: 10.1093/bioinformatics/btu638 25260700 PMC4287950

[B3] Belide S. Vanhercke T. Petrie J. R. Singh S. P. (2017). Robust genetic transformation of sorghum (Sorghum bicolor L.) using differentiating embryogenic callus induced from immature embryos. Plant Methods 13, 109. doi: 10.1186/s13007-017-0260-9 29234458 PMC5723044

[B4] Bidabadi S. S. Jain S. M. (2020). Cellular, molecular, and physiological aspects of *in vitro* plant regeneration. Plants 9, 702. doi: 10.3390/plants9060702 32492786 PMC7356144

[B5] Bürkle L. Cedzich A. Döpke C. Stransky H. Okumoto S. Gillissen B.. (2003). Transport of cytokinins mediated by purine transporters of the PUP family expressed in phloem, hydathodes, and pollen of Arabidopsis. Plant J. 34, 13–26. doi: 10.1046/j.1365-313X.2003.01700.x 12662305

[B6] Bustillo-Avendaño E. Ibáñez S. Sanz O. Sousa Barros J. A. Gude I. Perianez-Rodriguez J.. (2018). Regulation of hormonal control, cell reprogramming, and patterning during *de novo* root organogenesis. Plant Physiol. 176, 1709–1727. doi: 10.1104/pp.17.00980 29233938 PMC5813533

[B7] Chen Q. Sun J. Zhai Q. Zhou W. Qi L. Xu L.. (2011). The basic helix-loop-helix transcription factor MYC2 directly represses PLETHORA expression during jasmonate-mediated modulation of the root stem cell niche in arabidopsis. Plant Cell 23, 3335–3352. doi: 10.1105/tpc.111.089870 21954460 PMC3203420

[B8] Chen K. L. Xu M. X. Li G. Y. Liang H. Xia Z. L. Liu X.. (2006). Identification of AtENT3 as the main transporter for uridine uptake in Arabidopsis roots. Cell Res. 16, 377–388. doi: 10.1038/sj.cr.7310049 16617333

[B9] Cheng Z. J. Wang L. Sun W. Zhang Y. Zhou C. Su Y. H.. (2012). Pattern of auxin and cytokinin responses for shoot meristem induction results from the regulation of cytokinin biosynthesis by AUXIN RESPONSE FACTOR3. Plant Physiol. 161, 240–251. doi: 10.1104/pp.112.203166 23124326 PMC3532255

[B10] Cho H.-T. Cosgrove D. J. (2000). Altered expression of expansin modulates leaf growth and pedicel abscission in Arabidopsis thaliana. Proc. Natl. Acad. Sci. 97, 9783–9788. doi: 10.1073/pnas.160276997 10931949 PMC16942

[B11] Chung Y. Maharjan P. M. Lee O. Fujioka S. Jang S. Kim B.. (2011). Auxin stimulates DWARF4 expression and brassinosteroid biosynthesis in Arabidopsis. Plant J. 66, 564–578. doi: 10.1111/j.1365-313X.2011.04513.x 21284753

[B12] Chung Y. Zhu Y. Wu M.-F. Simonini S. Kuhn A. Armenta-Medina A.. (2019). Auxin Response Factors promote organogenesis by chromatin-mediated repression of the pluripotency gene SHOOTMERISTEMLESS. Nat. Commun. 10, 886. doi: 10.1038/s41467-019-08861-3 30792395 PMC6385194

[B13] Cordeiro D. Alves A. Ferraz R. Casimiro B. Canhoto J. Correia S. (2023). An efficient agrobacterium-mediated genetic transformation method for solanum betaceum cav. Embryogenic callus. Plants 12, 1202. doi: 10.3390/plants12051202 36904062 PMC10005457

[B14] Denis E. Kbiri N. Mary V. Claisse G. Conde e Silva N. Kreis M.. (2017). WOX14 promotes bioactive gibberellin synthesis and vascular cell differentiation in Arabidopsis. Plant J. 90, 560–572. doi: 10.1111/tpj.13513 28218997

[B15] Ding Z. Friml J. (2010). Auxin regulates distal stem cell differentiation in Arabidopsis roots. Proc. Natl. Acad. Sci. 107, 12046–12051. doi: 10.1073/pnas.1000672107 20543136 PMC2900669

[B16] Du X. Fang T. Liu Y. Huang L. Zang M. Wang G.. (2019). Transcriptome profiling predicts new genes to promote maize callus formation and transformation. Front. Plant Sci. 10. doi: 10.3389/fpls.2019.01633 PMC6934073 31921272

[B17] Fehér A. (2019). Callus, dedifferentiation, totipotency, somatic embryogenesis: what these terms mean in the era of molecular plant biology? Front. Plant Sci. 10. doi: 10.3389/fpls.2019.00536 PMC6524723 31134106

[B18] Frebort I. Kowalska M. Hluska T. Frebortova J. Galuszka P. (2011). Evolution of cytokinin biosynthesis and degradation. J. Exp. Bot. 62, 2431–2452. doi: 10.1093/jxb/err004 21321050

[B19] Gao M. Liu Y. Ma X. Shuai Q. Gai J. Li Y. (2017). Evaluation of reference genes for norMalization of gene expression using quantitative RT-PCR under aluminum, cadmium, and heat stresses in soybean. PLoS One 12, 1–15. doi: 10.1371/journal.pone.0168965 PMC5207429 28046130

[B20] Gillissen B. Bürkle L. André B. Kühn C. Rentsch D. Brandl B.. (2000). A new family of high-affinity transporters for adenine, cytosine, and purine derivatives in arabidopsis. Plant Cell 12, 291–300. doi: 10.1105/tpc.12.2.291 10662864 PMC139765

[B21] Goh T. Joi S. Mimura T. Fukaki H. (2012). The establishment of asymmetry in Arabidopsis lateral root founder cells is regulated by LBD16/ASL18 and related LBD/ASL proteins. Development 139, 883–893. doi: 10.1242/dev.071928 22278921

[B22] Goodstein D. M. Shu S. Howson R. Neupane R. Hayes R. D. Fazo J.. (2012). Phytozome: a comparative platform for green plant genomics. Nucleic Acids Res. 40, D1178–D1186. doi: 10.1093/nar/gkr944 22110026 PMC3245001

[B23] Grimplet J. Pimentel D. Agudelo-Romero P. Martinez-Zapater J. M. Fortes A. M. (2017). The LATERAL ORGAN BOUNDARIES Domain gene family in grapevine: genome-wide characterization and expression analyses during developmental processes and stress responses. Sci. Rep. 7, 15968. doi: 10.1038/s41598-017-16240-5 29162903 PMC5698300

[B24] Heberle H. Meirelles G. V. da Silva F. R. Telles G. P. Minghim R. (2015). InteractiVenn: a web-based tool for the analysis of sets through Venn diagrams. BMC Bioinf. 16, 169. doi: 10.1186/s12859-015-0611-3 PMC4455604 25994840

[B25] Hu X. Xu L. (2016). Transcription factors WOX11/12 directly activate WOX5/7 to promote root primordia initiation and organogenesis. Plant Physiol. 172, 2363–2373. doi: 10.1104/pp.16.01067 27784768 PMC5129711

[B26] Hwang H.-H. Wang C.-H. Chen H.-H. Ho J.-F. Chi S.-F. Huang F.-C.. (2019). Effective Agrobacterium-mediated transformation protocols for callus and roots of halophyte ice plant (Mesembryanthemum crystallinum). Bot. Stud. 60, 1. doi: 10.1186/s40529-018-0249-3 30617933 PMC6323063

[B27] Ikeuchi M. Favero D. S. Sakamoto Y. Iwase A. Coleman D. Rymen B.. (2019). Molecular mechanisms of plant regeneration. Annu. Rev. Plant Biol. 70, 377–406. doi: 10.1146/annurev-arplant-050718-100434 30786238

[B28] Ikeuchi M. Iwase A. Rymen B. Lambolez A. Kojima M. Takebayashi Y.. (2017). Wounding triggers callus formation via dynamic hormonal and transcriptional changes. Plant Physiol. 175, 1158–1174. doi: 10.1104/pp.17.01035 28904073 PMC5664475

[B29] Ikeuchi M. Sugimoto K. Iwase A. (2013). Plant callus: mechanisms of induction and repression. Plant Cell 25, 3159–3173. doi: 10.1105/tpc.113.116053 24076977 PMC3809525

[B30] Inzé D. De Veylder L. (2006). Cell cycle regulation in plant development. Annu. Rev. Genet. 40, 77–105. doi: 10.1146/annurev.genet.40.110405.090431 17094738

[B31] Iwase A. Mita K. Favero D. S. Mitsuda N. Sasaki R. Kobayshi M.. (2018). WIND1 induces dynamic metabolomic reprogramming during regeneration in Brassica napus. Dev. Biol. 442, 40–52. doi: 10.1016/j.ydbio.2018.07.006 30026120

[B32] Iwase A. Mitsuda N. Koyama T. Hiratsu K. Kojima M. Arai T.. (2011). The AP2/ERF transcription factor WIND1 controls cell dedifferentiation in arabidopsis. Curr. Biol. 21, 508–514. doi: 10.1016/j.cub.2011.02.020 21396822

[B33] Jiang N. Jeon E.-H. Pak J.-H. Ha T.-J. Baek I.-Y. Jung W.-S.. (2010). Increase of isoflavones in soybean callus by Agrobacterium-mediated transformation. Plant Biotechnol. Rep. 4, 253–260. doi: 10.1007/s11816-010-0143-2

[B34] Jin J. Tian F. Yang D.-C. Meng Y.-Q. Kong L. Luo J.. (2017). PlantTFDB 4.0: toward a central hub for transcription factors and regulatory interactions in plants. Nucleic Acids Res. 45, D1040–D1045. doi: 10.1093/nar/gkw982 27924042 PMC5210657

[B35] Kareem A. Durgaprasad K. Sugimoto K. Du Y. Pulianmackal A. J. Trivedi Z. B.. (2015). PLETHORA genes control regeneration by a two-step mechanism. Curr. Biol. 25, 1017–1030. doi: 10.1016/j.cub.2015.02.022 25819565 PMC4829346

[B36] Kim D. Paggi J. M. Park C. Bennett C. Salzberg S. L. (2019). Graph-based genome alignment and genotyping with HISAT2 and HISAT-genotype. Nat. Biotechnol. 37, 907–915. doi: 10.1038/s41587-019-0201-4 31375807 PMC7605509

[B37] Ko D. Kang J. Kiba T. Park J. Kojima M. Do J.. (2014). Arabidopsis ABCG14 is essential for the root-to-shoot translocation of cytokinin. Proc. Natl. Acad. Sci. 111, 7150–7155. doi: 10.1073/pnas.1321519111 24778257 PMC4024864

[B38] Kolde R. (2015) pheatmap: Pretty Heatmaps. R Packag. version 1.0.8. Available at: https://cran.r-project.org/web/packages/pheatmap/pheatmap.pdf.

[B39] Kong D. Hao Y. Cui H. (2016). The WUSCHEL related homeobox protein WOX7 regulates the sugar response of lateral root development in arabidopsis thaliana. Mol. Plant 9, 261–270. doi: 10.1016/j.molp.2015.11.006 26621542

[B40] Kowalczyk M. Sandberg G. (2001). Quantitative analysis of indole-3-acetic acid metabolites in arabidopsis. Plant Physiol. 127, 1845–1853. doi: 10.1104/pp.010525 11743128 PMC133588

[B41] Kumar L. Futschik M. E. (2007). Mfuzz: A software package for soft clustering of microarray data. Bioinformation 2, 5–7. doi: 10.6026/97320630002005 18084642 PMC2139991

[B42] Lee H. W. Kim M.-J. Kim N. Y. Lee S. H. Kim J. (2013). LBD18 acts as a transcriptional activator that directly binds to the EXPANSIN14 promoter in promoting lateral root emergence of Arabidopsis. Plant J. 73, 212–224. doi: 10.1111/tpj.12013 22974309

[B43] Lee K. Seo P. J. (2017). High-temperature promotion of callus formation requires the BIN2-ARF-LBD axis in Arabidopsis. Planta 246, 797–802. doi: 10.1007/s00425-017-2747-z 28766014

[B44] Li S.-B. Xie Z.-Z. Hu C.-G. Zhang J.-Z. (2016). A review of auxin response factors (ARFs) in plants. Front. Plant Sci. 7. doi: 10.3389/fpls.2016.00047 PMC4737911 26870066

[B45] Liu Z. Li J. Wang L. Li Q. Lu Q. Yu Y.. (2016). Repression of callus initiation by the miRNA-directed interaction of auxin–cytokinin in Arabidopsis thaliana. Plant J. 87, 391–402. doi: 10.1111/tpj.13211 27189514

[B46] Liu W. Yu J. Ge Y. Qin P. Xu L. (2018). Pivotal role of LBD16 in root and root-like organ initiation. Cell. Mol. Life Sci. 75, 3329–3338. doi: 10.1007/s00018-018-2861-5 29943076 PMC11105430

[B47] Love M. I. Huber W. Anders S. (2014). Moderated estimation of fold change and dispersion for RNA-seq data with DESeq2. Genome Biol. 15, 550. doi: 10.1186/s13059-014-0550-8 25516281 PMC4302049

[B48] Oüstin A. Kowalyczk M. Bhalerao R. P. Sandberg G. (1998). Metabolism of indole-3-acetic acid in arabidopsis1. Plant Physiol. 118, 285–296. doi: 10.1104/pp.118.1.285 9733548 PMC34867

[B49] Oh M.-H. Honey S. H. Tax F. E. (2020). The control of cell expansion, cell division, and vascular development by brassinosteroids: A historical perspective. Int. J. Mol. Sci. 21, 1743. doi: 10.3390/ijms21051743 32143305 PMC7084555

[B50] Okushima Y. Fukaki H. Onoda M. Theologis A. Tasaka M. (2007). ARF7 and ARF19 regulate lateral root formation via direct activation of LBD/ASL genes in arabidopsis. Plant Cell 19, 118–130. doi: 10.1105/tpc.106.047761 17259263 PMC1820965

[B51] Pitaksaringkarn W. Matsuoka K. Asahina M. Miura K. Sage-Ono K. Ono M.. (2014). XTH20 and XTH19 regulated by ANAC071 under auxin flow are involved in cell proliferation in incised Arabidopsis inflorescence stems. Plant J. 80, 604–614. doi: 10.1111/tpj.12654 25182467

[B52] Qi F. Zhang F. (2020). Cell cycle regulation in the plant response to stress. Front. Plant Sci. 10. doi: 10.3389/fpls.2019.01765 PMC7002440 32082337

[B53] Rane R. V. Oakeshott J. G. Nguyen T. Hoffmann A. A. Lee S. F. (2017). Orthonome – a new pipeline for predicting high quality orthologue gene sets applicable to complete and draft genomes. BMC Genomics 18, 673. doi: 10.1186/s12864-017-4079-6 28859620 PMC5580312

[B54] Rao X. Huang X. Zhou Z. Lin X. (2013). An improvement of the 2ˆ(-delta CT) method for quantitative real-time polymerase chain reaction data analysis. Biostat. Bioinforma. Biomath. 3, 71–85. 25558171 PMC4280562

[B55] Sairam R. V. Franklin G. Hassel R. Smith B. Meeker K. Kashikar N.. (2003). A study on the effect of genotypes, plant growth regulators and sugars in promoting plant regeneration via organogenesis from soybean cotyledonary nodal callus. Plant Cell. Tissue Organ Cult. 75, 79–85. doi: 10.1023/A:1024649122748

[B56] Sakakibara H. (2010). “Cytokinin biosynthesis and metabolism,” in Plant hormones (Dordrecht: Springer Netherlands), 95–114. doi: 10.1007/978-1-4020-2686-7_5

[B57] Sanchez Carranza A. P. Singh A. Steinberger K. Panigrahi K. Palme K. Dovzhenko A.. (2016). Hydrolases of the ILR1-like family of Arabidopsis thaliana modulate auxin response by regulating auxin homeostasis in the endoplasmic reticulum. Sci. Rep. 6, 24212. doi: 10.1038/srep24212 27063913 PMC4827090

[B58] Schmutz J. Cannon S. B. Schlueter J. Ma J. Mitros T. Nelson W.. (2010). Genome sequence of the palaeopolyploid soybean. Nature 463, 178–183. doi: 10.1038/nature08670 20075913

[B59] Shen S. Sun F. Zhu M. Chen S. Guan M. Chen R.. (2020). Genome-wide identification AINTEGUMENTA-like (AIL) genes in Brassica species and expression patterns during reproductive development in Brassica napus L. PLoS One 15, e0234411. doi: 10.1371/journal.pone.0234411 32511257 PMC7279594

[B60] Shimotohno A. Aki S. S. Takahashi N. Umeda M. (2021). Regulation of the plant cell cycle in response to hormones and the environment. Annu. Rev. Plant Biol. 72, 273–296. doi: 10.1146/annurev-arplant-080720-103739 33689401

[B61] Skirycz A. Radziejwoski A. Busch W. Hannah M. A. Czeszejko J. Kwaśniewski M.. (2008). The DOF transcription factor OBP1 is involved in cell cycle regulation in Arabidopsis thaliana. Plant J. 56, 779–792. doi: 10.1111/j.1365-313X.2008.03641.x 18665917

[B62] Skoog F. Miller C. O. (1957). Chemical regulation of growth and organ formation in plant tissues cultured *in vitro* . Symp. Soc Exp. Biol. 11, 118–130. 13486467

[B63] Sugimoto K. Jiao Y. Meyerowitz E. M. (2010). Arabidopsis regeneration from multiple tissues occurs via a root development pathway. Dev. Cell 18, 463–471. doi: 10.1016/j.devcel.2010.02.004 20230752

[B64] Sun J. Hirose N. Wand X. Web P. Xue L. Sakakibara H.. (2005). Arabidopsis SOI33/atENT8 gene encodes a putative equilibrative nucleoside transporter that is involved in cytokinin transport in planta. J. Integr. Plant Biol. 47, 588–603. doi: 10.1111/j.1744-7909.2005.00104.x

[B65] Sun J. Li L. Wang P. Zhang S. Wu J. (2017). Genome-wide characterization, evolution, and expression analysis of the leucine-rich repeat receptor-like protein kinase (LRR-RLK) gene family in Rosaceae genomes. BMC Genomics 18, 763. doi: 10.1186/s12864-017-4155-y 29017442 PMC5635495

[B66] Suo J. Zhou C. Zeng Z. Li X. Bian H. Wang J.. (2021). Identification of regulatory factors promoting embryogenic callus formation in barley through transcriptome analysis. BMC Plant Biol. 21, 145. doi: 10.1186/s12870-021-02922-w 33740900 PMC7980361

[B67] Thibaud-Nissen F. Shealy R. T. Khanna A. Vodkin L. O. (2003). Clustering of microarray data reveals transcript patterns associated with somatic embryogenesis in soybean. Plant Physiol. 132, 118–136. doi: 10.1104/pp.103.019968 12746518 PMC166958

[B68] Tian F. Yang D.-C. Meng Y.-Q. Jin J. Gao G. (2019). PlantRegMap: charting functional regulatory maps in plants. Nucleic Acids Res. 48, D1104–D1113. doi: 10.1093/nar/gkz1020 PMC7145545 31701126

[B69] Tokunaga H. Kojima M. Kuroha T. Ishida T. Sugimoto K. Kiba T.. (2012). Arabidopsis lonely guy (LOG) multiple mutants reveal a central role of the LOG-dependent pathway in cytokinin activation. Plant J. 69, 355–365. doi: 10.1111/j.1365-313X.2011.04795.x 22059596

[B70] Wang L. Cao C. Ma Q. Zeng Q. Wang H. Cheng Z.. (2014). RNA-seq analyses of multiple meristems of soybean: novel and alternative transcripts, evolutionary and functional implications. BMC Plant Biol. 14, 169. doi: 10.1186/1471-2229-14-169 24939556 PMC4070088

[B71] Wei Z. Li J. (2016). Brassinosteroids regulate root growth, development, and symbiosis. Mol. Plant 9, 86–100. doi: 10.1016/j.molp.2015.12.003 26700030

[B72] Wu T. Hu E. Xu S. Chen M. Guo P. Dai Z.. (2021). clusterProfiler 4.0: A universal enrichment tool for interpreting omics data. Innov 2, 100141. doi: 10.1016/j.xinn.2021.100141 PMC8454663 34557778

[B73] Xu C. Cao H. Xu E. Zhang S. Hu Y. (2018). Genome-Wide Identification of Arabidopsis LBD29 Target Genes Reveals the Molecular Events behind Auxin-Induced Cell Reprogramming during Callus Formation. Plant Cell Physiol. 59, 749–760. doi: 10.1093/pcp/pcx168 29121271

[B74] Yoon Y. Seo D. H. Shin H. Kim H. J. Kim C. M. Jang G. (2020). The role of stress-responsive transcription factors in modulating abiotic stress tolerance in plants. Agronomy 10, 788. doi: 10.3390/agronomy10060788

[B75] Zhang A. Matsuoka K. Kareem A. Robert M. Roszak P. Blob B.. (2022). Cell-wall damage activates DOF transcription factors to promote wound healing and tissue regeneration in Arabidopsis thaliana. Curr. Biol. 32, 1883–1894.e7. doi: 10.1016/j.cub.2022.02.069 35320706

