# Supplementary figures and images for: Uncovering transcriptional reprogramming during callus development in soybean: insights and implications
Source: Front Plant Sci. 2023 Aug 4;14:1239917. doi: 10.3389/fpls.2023.1239917 (PMC10436568; doi:10.3389/fpls.2023.1239917)

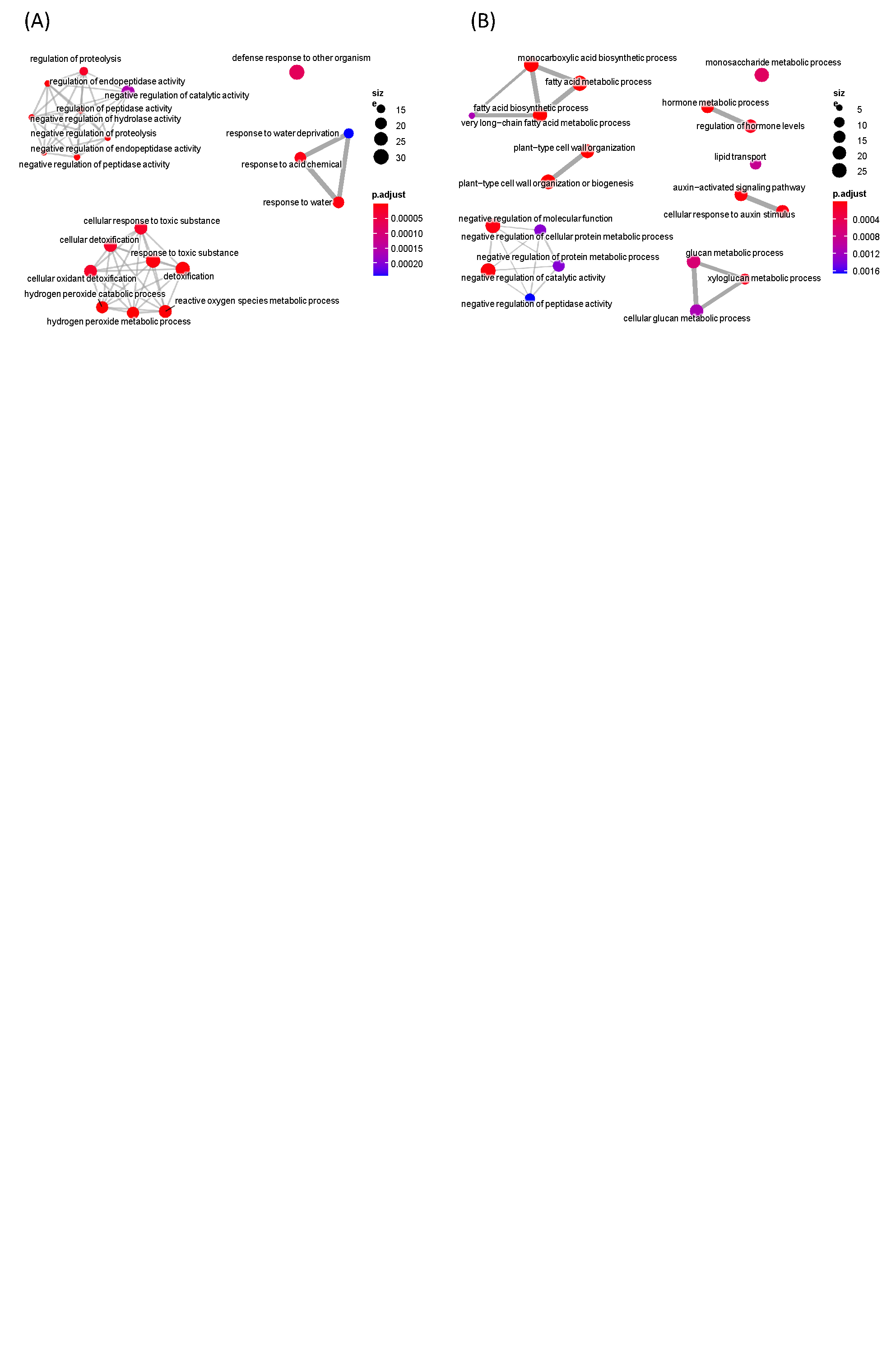

Supplement: Supplementary Figure 1 — Summary of similarity among samples. The enrichment maps show the top 20 enriched biological processes based on the analysis of top 1000 genes in PC1 (A) and PC2 (B), respectively. [file Image_1.tif]

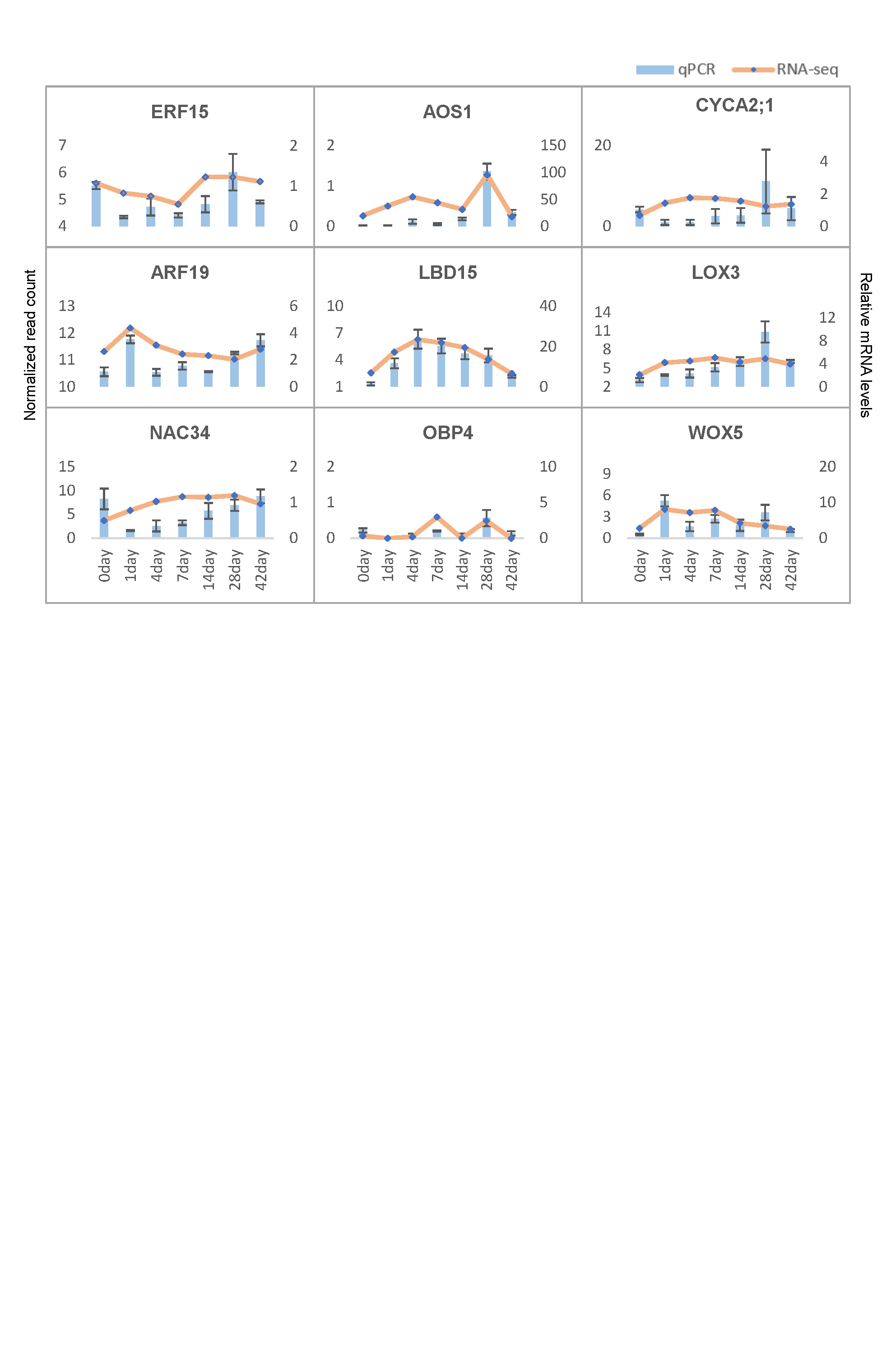

Supplement: Supplementary Figure 2 — Confirmation of RNA sequencing results using real-time PCR analysis. The expression values obtained from RNA-seq were validated by real-time PCR analysis. The left and right scales represent RNA-Seq and qRT-PCR values, respectively. Error bars indicate the standard error calculated from three biological replicates and three technical replicates. ERF15 (Glyma.01G206600); AOS1 (Glyma.04G035000); CYCA2;1 (Glyma.04G071600); ARF19 (Glyma.09G072200); LBD15 (Glyma.03G161500); LOX3 (Glyma.03G264300); OBP4 (Glyma.05G018100); NAC34 (Glyma.08G173400); WOX5 (Glyma.02G254800). [file Image_2.tif]
